# Antagonism of STAT3 signalling by Ebola virus

**DOI:** 10.1371/journal.ppat.1009636

**Published:** 2021-06-24

**Authors:** Angela R. Harrison, Shawn Todd, Megan Dearnley, Cassandra T. David, Diane Green, Stephen M. Rawlinson, Gough G. Au, Glenn A. Marsh, Gregory W. Moseley

**Affiliations:** 1 Department of Microbiology, Monash Biomedicine Discovery Institute, Monash University, Clayton, Victoria, Australia; 2 Australian Centre for Disease Preparedness, CSIRO, Geelong, Victoria, Australia; University of Texas Medical Branch / Galveston National Laboratory, UNITED STATES

## Abstract

Many viruses target signal transducers and activators of transcription (STAT) 1 and 2 to antagonise antiviral interferon signalling, but targeting of signalling by other STATs/cytokines, including STAT3/interleukin 6 that regulate processes important to Ebola virus (EBOV) haemorrhagic fever, is poorly defined. We report that EBOV potently inhibits STAT3 responses to interleukin-6 family cytokines, and that this is mediated by the interferon-antagonist VP24. Mechanistic analysis indicates that VP24 effects a unique strategy combining distinct karyopherin-dependent and karyopherin-independent mechanisms to antagonise STAT3-STAT1 heterodimers and STAT3 homodimers, respectively. This appears to reflect distinct mechanisms of nuclear trafficking of the STAT3 complexes, revealed for the first time by our analysis of VP24 function. These findings are consistent with major roles for global inhibition of STAT3 signalling in EBOV infection, and provide new insights into the molecular mechanisms of STAT3 nuclear trafficking, significant to pathogen-host interactions, cell physiology and pathologies such as cancer.

## Author summary

Ebola virus (EBOV) continues to pose a significant risk to human health globally, causing ongoing disease outbreaks with case-fatality rates between 40 and 65%. Suppression of immune responses is a critical component of EBOV haemorrhagic fever, but understanding of EBOV impact on signalling by cytokines other than interferon is limited. We find that infectious EBOV inhibits interleukin-6 cytokine signalling *via* antagonism of STAT3. The antagonistic strategy uniquely combines two distinct mechanisms, which appear to reflect differing nuclear trafficking mechanisms of critical STAT3 complexes. This provides fundamental insights into the mechanisms of pathogenesis of a lethal virus, and biology of STAT3, a critical player in immunity, development, growth and cancer.

## Introduction

Outbreaks of Ebola virus (EBOV, species *Zaire ebolavirus*, family *Filoviridae*, order *Mononegavirales*) cause severe haemorrhagic fever with fatality rates in recent outbreaks between 40 and 65% [1–4]. The 2014–2016 West African outbreak (> 11,000 human deaths), and recent outbreak in the Democratic Republic of Congo (c. 2300 deaths in 2018–2020) highlight the ongoing danger to human health [3,4].

The capacity of mammalian viruses to overcome the type-I interferon (IFN)-mediated antiviral innate immune response is an important factor in virulence [5–7]. IFNs are induced in response to cellular detection of viral infection, and signal in autocrine and paracrine fashion to activate intracellular signalling, principally through STAT1 and STAT2. Following IFN-receptor binding, STAT1/2 are phosphorylated at conserved tyrosines, which results in the formation of phospho-(pY-)STAT1-STAT2 heterodimers and pY-STAT1 homodimers. Nuclear localisation signals (NLSs) formed within the dimers bind to nuclear import receptors of the NPI-1 karyopherin subfamily (which include karyopherin alpha-1 (Kα1)) at a ‘non-classical’ cargo-binding site, distinct from sites bound by most cellular cargoes [8–10]. The karyopherins mediate active nuclear accumulation of the STAT dimers, leading to antiviral IFN-stimulated gene (ISG) activation [11]. To evade IFN-dependent immune signalling, viruses encode IFN-antagonist proteins, many of which target STAT1/STAT2, including through interactions leading to sequestration, induction of degradation and inhibition of phosphorylation [5]. Among IFN-antagonists, EBOV VP24 uses an unusual mechanism of competitive binding at the non-classical STAT1-binding site in NPI-1 karyopherins, thereby preventing STAT1 nuclear trafficking and ISG induction [12–15].

While IFN-STAT1/2 antagonism is reasonably well understood for many viruses, antagonism of other STATs including STAT3, the major mediator of signalling by interleukin (IL)-6 family cytokines (e.g. IL-6, oncostatin-M (OSM) [11]), is poorly defined, with a limited number of mononegaviruses (three paramyxoviruses and several members of the genus *Lyssavirus*) shown to express IFN-antagonist proteins that interact with STAT3 [16–20]. Nevertheless, STAT3-regulated processes are strongly implicated/dysregulated in EBOV disease, including the pro-inflammatory response, coagulation pathway and wound healing [6,21–23]. Notably, despite critical roles in processes such as growth, development, apoptosis, infection and cancer, the precise mechanism(s) underlying cytokine-dependent STAT3 nuclear accumulation also remain poorly understood. Contrasting reports suggest three models whereby: (i) STAT3 undergoes constitutive nucleocytoplasmic shuttling by karyopherins including Kα4, with cytokines inducing intra-nuclear sequestration [24,25], (ii) cytokine activation induces interaction of STAT3 with karyopherins including Kα1 resulting in nuclear import similar to STAT1 [26,27], and (iii) STAT3 uses a combination of these mechanisms [28]. Notably, pY-STAT3 forms homodimers as well as heterodimers with pY-STAT1, which may regulate distinct gene subsets [20,29] and could use different trafficking mechanisms, possibly accounting for the contrasting models; this has not been directly examined.

Here, we aimed to examine the effect of EBOV on STAT3 responses, showing for the first time that EBOV VP24 antagonises STAT3 using a combination of mechanisms analogous to and distinct from that used for STAT1, to inhibit both STAT3 homodimers and heterodimers. We further reveal that the STAT3 complexes use distinct mechanisms for nuclear accumulation, apparently necessitating VP24’s multipronged strategy.

## Results and discussion

### EBOV inhibits STAT3 responses

**Fig 1:**
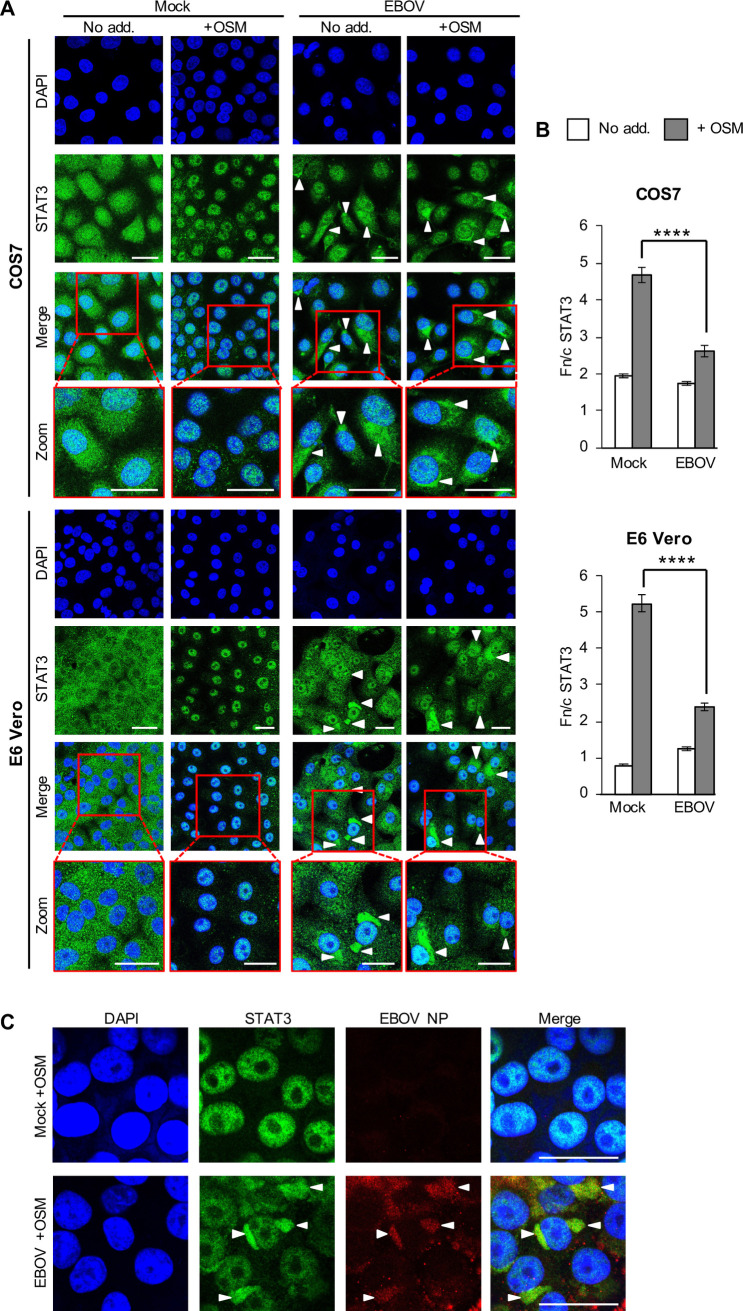
EBOV infection inhibits STAT3 responses to OSM. (A) COS7 (upper panel) or E6 Vero (lower panel) cells infected with EBOV (MOI 10, which results in infection of c. 100% of cells, see S1 Fig) or mock-infected were treated 72 h post-infection with or without OSM (10 ng/ml, 15 min) before fixation, immunofluorescent staining for STAT3 (green), and analysis by CLSM. DAPI (blue) was used to localise nuclei. Representative images are shown. Arrowheads indicate accumulation of STAT3 in cytoplasmic regions; indicated regions in merged images are expanded in panels below (Zoom). (B) Images such as those shown in A were analysed to calculate the nuclear to cytoplasmic fluorescence ratio (Fn/c) for STAT3 (mean ± SEM, n ≥ 70 cells for each condition). Statistical analysis (Student’s *t*-test) was performed using GraphPad Prism software; ****, p < 0.0001; No add., no addition. (C) E6 Vero cells were infected and treated as in A, before fixation, immunofluorescent staining for STAT3 (green) and EBOV NP (using monoclonal anti-NP, red), and analysis by CLSM. DAPI (blue) was used to localise nuclei. Representative images are shown. Arrowheads indicate colocalization of STAT3 and NP in discrete cytoplasmic regions/inclusions. Scale bars, 30 μm.

Despite likely roles in EBOV infection for dysregulation of cytokines/STATs other than IFN/STAT1/2, antagonism of other STATs by EBOV remains unresolved. To determine whether EBOV affects STAT3, we infected COS7 cells with EBOV before treatment with OSM, commonly used to analyse IL-6 family cytokine/STAT3 responses [18,20,26], and analysis of STAT3 localisation by immunofluorescence staining and confocal laser scanning microscopy (CLSM; Fig 1A). In mock-infected cells, STAT3 was diffusely localised between the nucleus and cytoplasm of resting cells, with nuclear accumulation clearly observed following OSM treatment, as expected. In EBOV-infected cells, however, OSM-dependent STAT3 nuclear accumulation was inhibited, with quantitative image analysis confirming a significant decrease in nucleocytoplasmic localisation in EBOV-infected compared with mock-infected cells (Fig 1A and 1B). To exclude possible impact by virus-induced type-I IFN, we confirmed that EBOV also antagonises STAT3 responses in Vero cells, which do not produce IFN (Fig 1A and 1B). Notably, in infected cells, we observed accumulation of STAT3 into large, distinct cytoplasmic regions (zoom images, Fig 1A). Co-staining for EBOV nucleoprotein (NP) using different monoclonal (Fig 1C) or polyclonal (S1 Fig) antibodies indicated that these regions correspond to cytoplasmic viral replication/inclusion bodies [30]. This concentration/colocalization of STAT3 with cytoplasmic viral inclusions is, to our knowledge, the first such observation for any virus, and suggests that STAT3 is accumulated into these virus-induced structures.

### Stimulation of cells with OSM before infection inhibits EBOV replication

**Fig 2:**
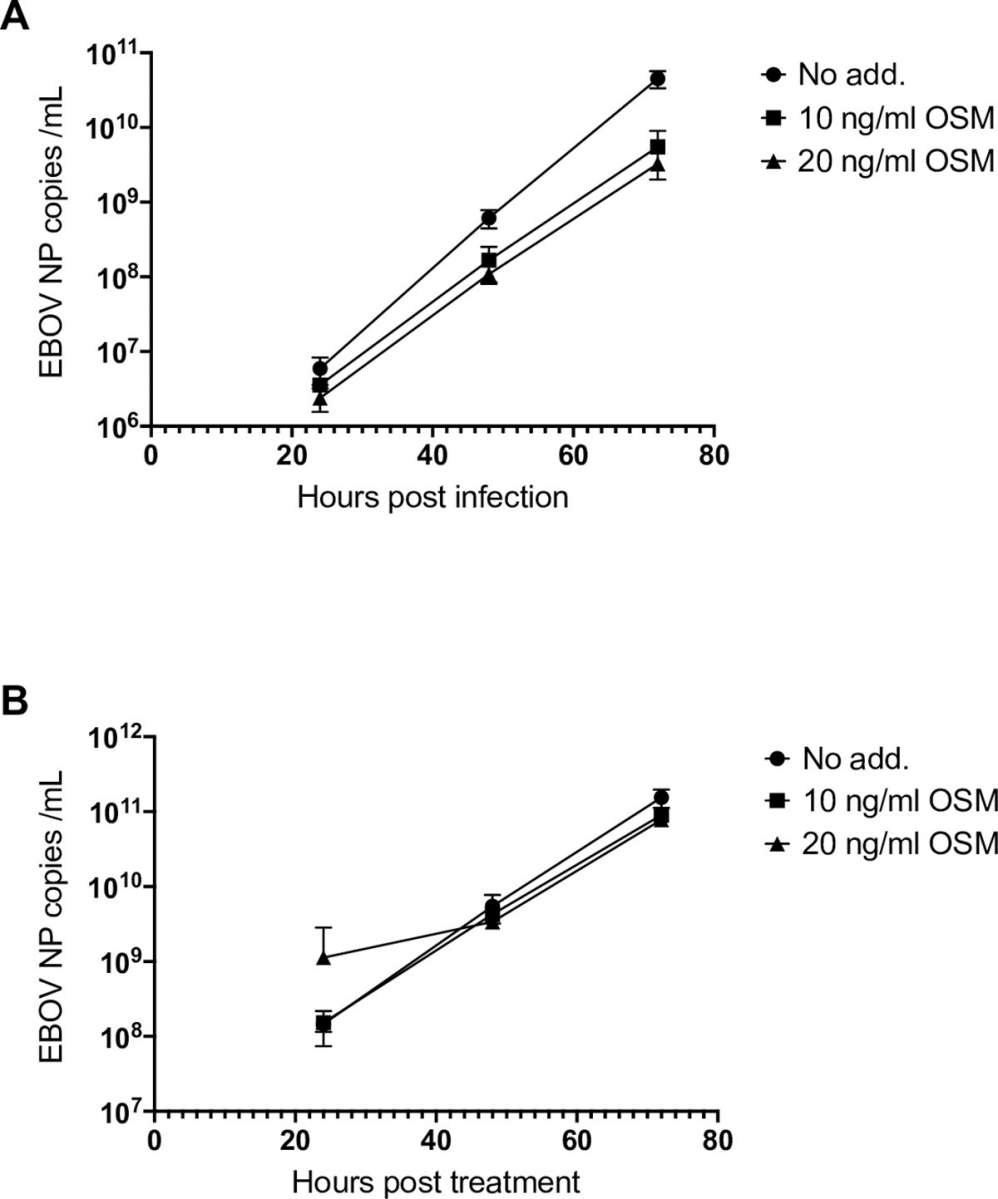
EBOV replication is inhibited by treatment of cells with OSM before infection. E6 Vero cells were treated without or with the indicated concentration of OSM for 24 h before infection with EBOV (MOI 1) for 24 h, 48 h and 72 h (A), or were infected (MOI 1) 24 h before treatment without or with OSM for 24 h, 48 h and 72 h (B). Virus was quantified by RT-qPCR for EBOV NP. Data show mean ± SD; n = 3 biological replicates.

The finding that EBOV inhibits STAT3 responses suggests that STAT3-dependent cytokine signalling is likely to have antiviral functions toward EBOV, analogous to reports for hepatitis C virus [31], herpes simplex virus-1 [32], coxsackievirus B3 [33], influenza virus and vaccinia virus [34]. To examine this, we assessed the effect of OSM stimulation of Vero cells on EBOV replication by treatment of cells with OSM 24 h pre-infection or 24 h post-infection. Analysis using RT-qPCR for EBOV NP [35] indicated that OSM treatment pre-infection inhibits replication (Fig 2A). This suggests that OSM/STAT3 signalling can induce an antiviral state which impedes subsequent viral infection, resulting in a lag in replication. No comparable effect was detected following stimulation of cells after establishment of infection (Fig 2B), consistent with antagonism of antiviral signalling by EBOV, resulting in a less potent effect of OSM on replication.

### EBOV VP24 antagonises OSM/STAT3 signalling

**Fig 3:**
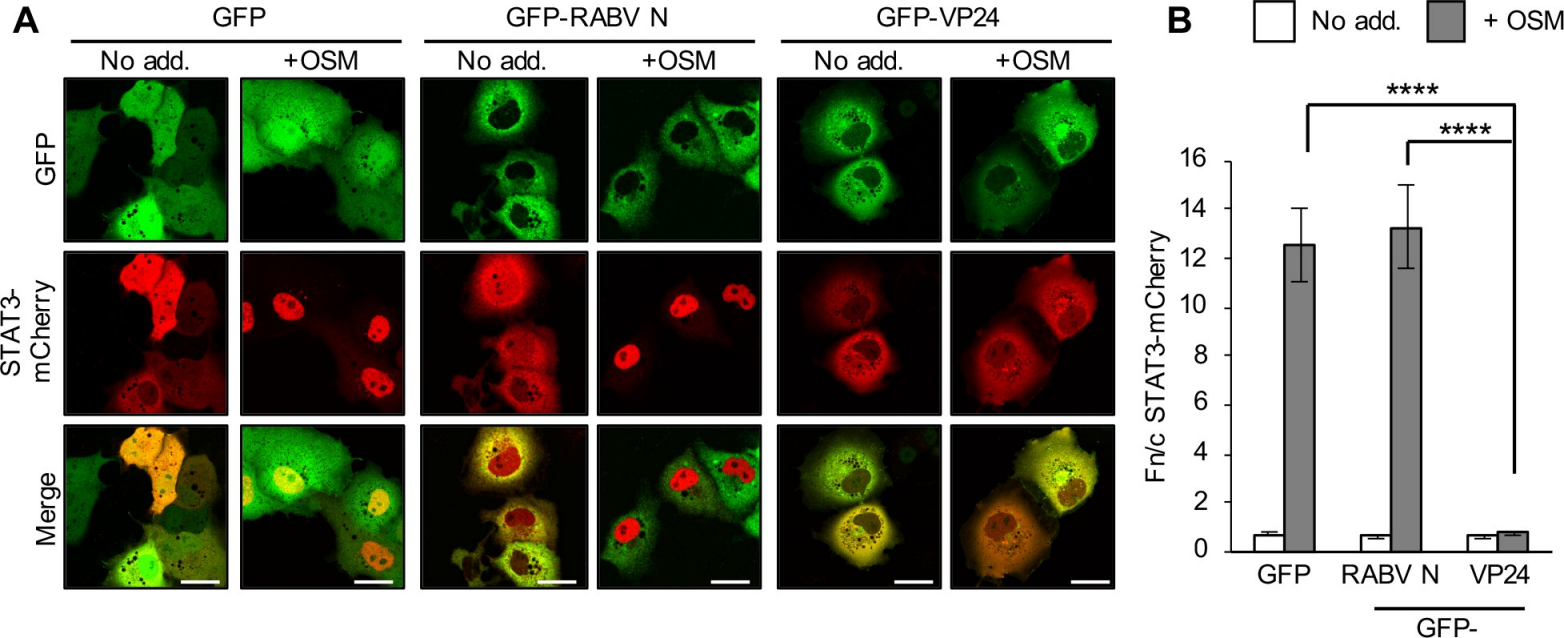
EBOV VP24 protein expression inhibits STAT3 responses to OSM. COS7 cells co-transfected to express the indicated proteins were treated 24 h post-transfection with or without OSM (10 ng/ml, 30 min) before live-cell CLSM analysis (A) to determine the Fn/c for STAT3-mCherry (B; mean ± SEM; n ≥ 35 cells for each condition; results are from a single assay representative of two independent assays). Scale bars, 30 μm. RABV N, rabies virus N-protein. Statistical analysis used Student’s *t*-test; ****, p < 0.0001.

**Fig 4:**
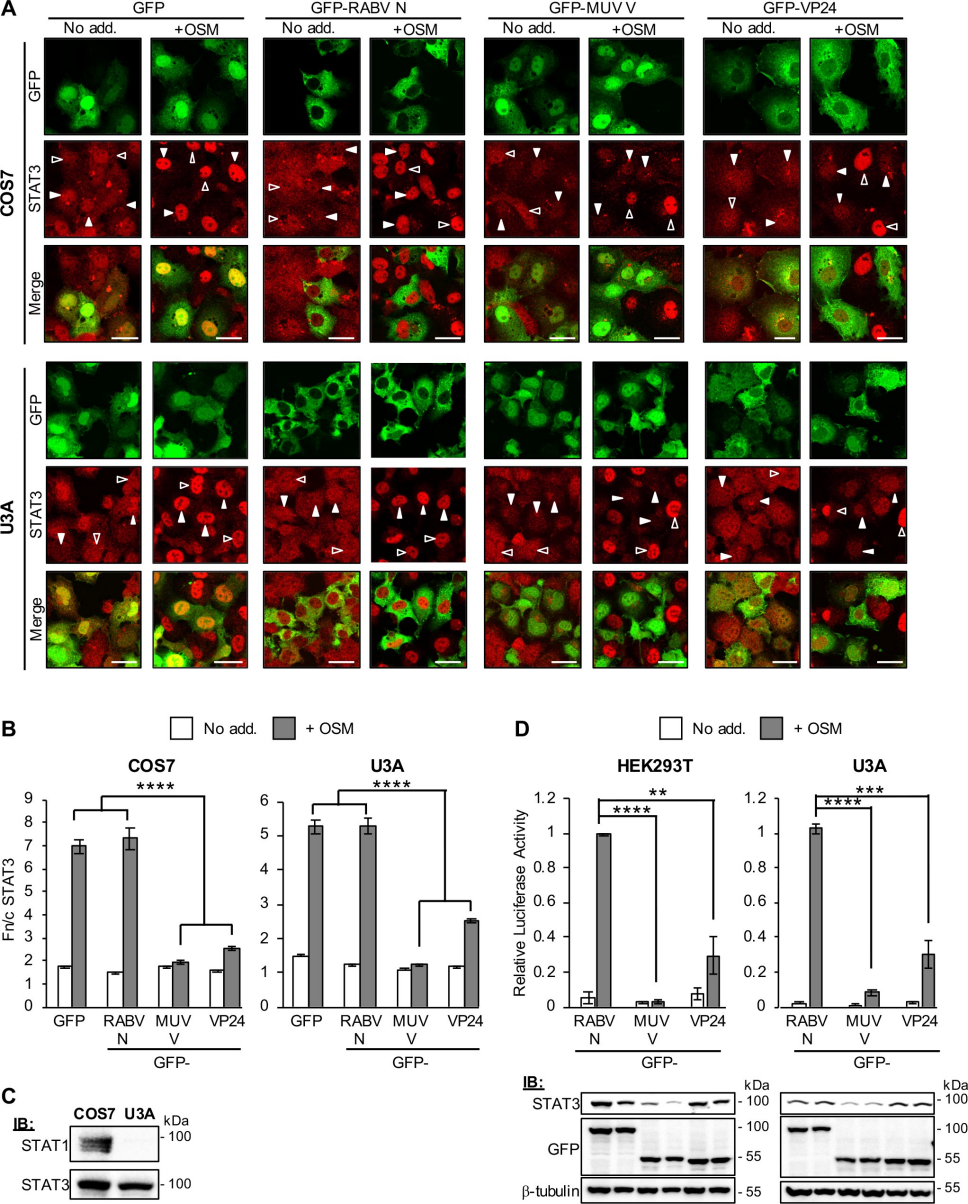
EBOV VP24 antagonises STAT3 independently of STAT1. (A, B) COS7 (upper panel) or U3A (lower panel) cells transfected to express the indicated proteins were treated 24 h post-transfection with or without OSM (10 ng/ml, 15 min) before fixation, immunofluorescent staining for STAT3 (red) and CLSM (A) to determine the Fn/c for STAT3 (B; mean ± SEM, n ≥ 36 cells for each condition; results are from a single assay representative of two independent assays). Filled and unfilled arrowheads indicate cells with or without, respectively, detectable expression of the transfected protein. Scale bars, 30 μm. MUV V, Mumps virus V protein. (C) Lysates of COS7 and U3A cells were analysed by immunoblotting (IB) for STAT1 and STAT3. (D) *upper panel*: HEK293T or U3A cells co-transfected with m67-LUC and pRL-TK plasmids, and plasmids to express the indicated proteins, were treated 16 h post-transfection with or without OSM (10 ng/ml, 8 h) before determination of relative luciferase activity (mean ± SEM; n = 3 independent assays); *lower panel*: cell lysates used in a representative assay were analysed by IB using antibodies against the indicated proteins. Statistical analysis used Student’s *t*-test; **, p<0.01; ***, p < 0.001; ****, p < 0.0001.

Since VP24 antagonises IFN/STAT1 responses [12], we tested its effects on STAT3 by analysing COS7 cells expressing GFP-VP24 or negative controls (GFP or GFP-rabies virus (RABV) N-protein, which does not affect STAT3 [18,20]), and co-transfected to express mCherry-STAT3 (for live-cell analysis; Fig 3) or immunostained for endogenous STAT3 (Fig 4A and 4B). OSM effected clear nuclear accumulation of STAT3 in GFP and RABV N-protein-expressing cells, but this was strongly inhibited in VP24-expressing cells. VP24 also potently inhibited STAT3 responses in HeLa and Vero cells (S2 Fig), similar to observations in infected Vero cells, indicating that antagonism of STAT3 is independent of IFN. Since OSM can induce pY-STAT3 homodimers and pY-STAT3-pY-STAT1 heterodimers [36] and VP24 antagonises pY-STAT1 [12], we assessed the dependence of VP24-STAT3 antagonism on STAT1 using STAT1-deficient U3A cells [37,38]. VP24 clearly antagonised STAT3 in U3A cells (Fig 4A and 4B) in which we confirmed a lack of STAT1 expression (Fig 4C), indicating that VP24 can inhibit STAT3 independently of STAT1 and thus target STAT3 homodimers.

The above data clearly indicated that VP24 has autonomous activity in antagonising STAT3 responses, independent of other viral components. However, our observation that STAT3 appears to accumulate into NP-enriched inclusion bodies in EBOV-infected cells (Fig 1), combined with the observation that VP24 and NP interact [39], suggested that NP/inclusions might enhance or otherwise regulate VP24-mediated antagonism. We thus assessed STAT3 responses to OSM in cells expressing VP24 with and without NP (S3 Fig). In OSM-treated cells expressing NP and GFP (control), STAT3 retained clear capacity to accumulate into the nucleus, and this did not differ significantly from cells expressing GFP alone. In cells expressing VP24, STAT3 responses were significantly inhibited to a similar extent in cells expressing or not expressing NP. These data confirm a principal role for VP24 in antagonism of STAT3, and indicate that NP does not significantly contribute to (or impede) this function. Thus, VP24 is sufficient to effect STAT3 antagonism in the absence of NP or viral infection. However, given the apparent accumulation of STAT3 into large cytoplasmic viral inclusions in infected cells (Fig 1), we cannot rule out that this contributes to efficient STAT3 antagonism by VP24 during infection, potentially involving other viral components. Our observations in this respect are analogous to reports of sequestration of STAT1, STAT2 and other antiviral signalling molecules (e.g. TANK-binding kinase 1, IFN regulatory factor 3) in cytoplasmic inclusions formed by other negative-sense RNA viruses [16,17,40,41]. However, given the diverse functions attributed to STAT3, including a number of reports that STAT3 can have pro-viral rather than antiviral functions depending on the specific virus, cell type and other parameters [42], it is possible that recruitment of STAT3 to inclusion bodies plays distinct roles in EBOV infection. These possibilities will form the basis of future research.

To confirm the functional outcome of VP24-mediated inhibition of STAT3 nuclear accumulation, we next analysed OSM-dependent signalling using a luciferase reporter gene assay [18,43], in which the luciferase gene is under the control of a STAT3-dependent promoter (m67). This indicated that VP24 effects significant suppression (c. 70% reduction) of OSM/STAT3 signalling in HEK293T and U3A cells (Fig 4D; upper panel); RT-qPCR analysis confirmed that VP24 can inhibit OSM-induced expression of the STAT3-dependent *socs3* gene (S4 Fig). Mumps virus V-protein (MUV-V, used as a positive control in our assays) induces STAT3 degradation to suppress IL-6 signalling [16]. We confirmed that MUV-V inhibits STAT3 responses and that this correlates with reduced levels of STAT3 expression in cell lysates. Since no similar effect was observed on STAT3 expression in VP24-expressing cells (Fig 4D; lower panel), it appeared that VP24 uses a different antagonistic mechanism.

### VP24 inhibits Kα1 interaction with STAT3, dependent on STAT1

**Fig 5:**
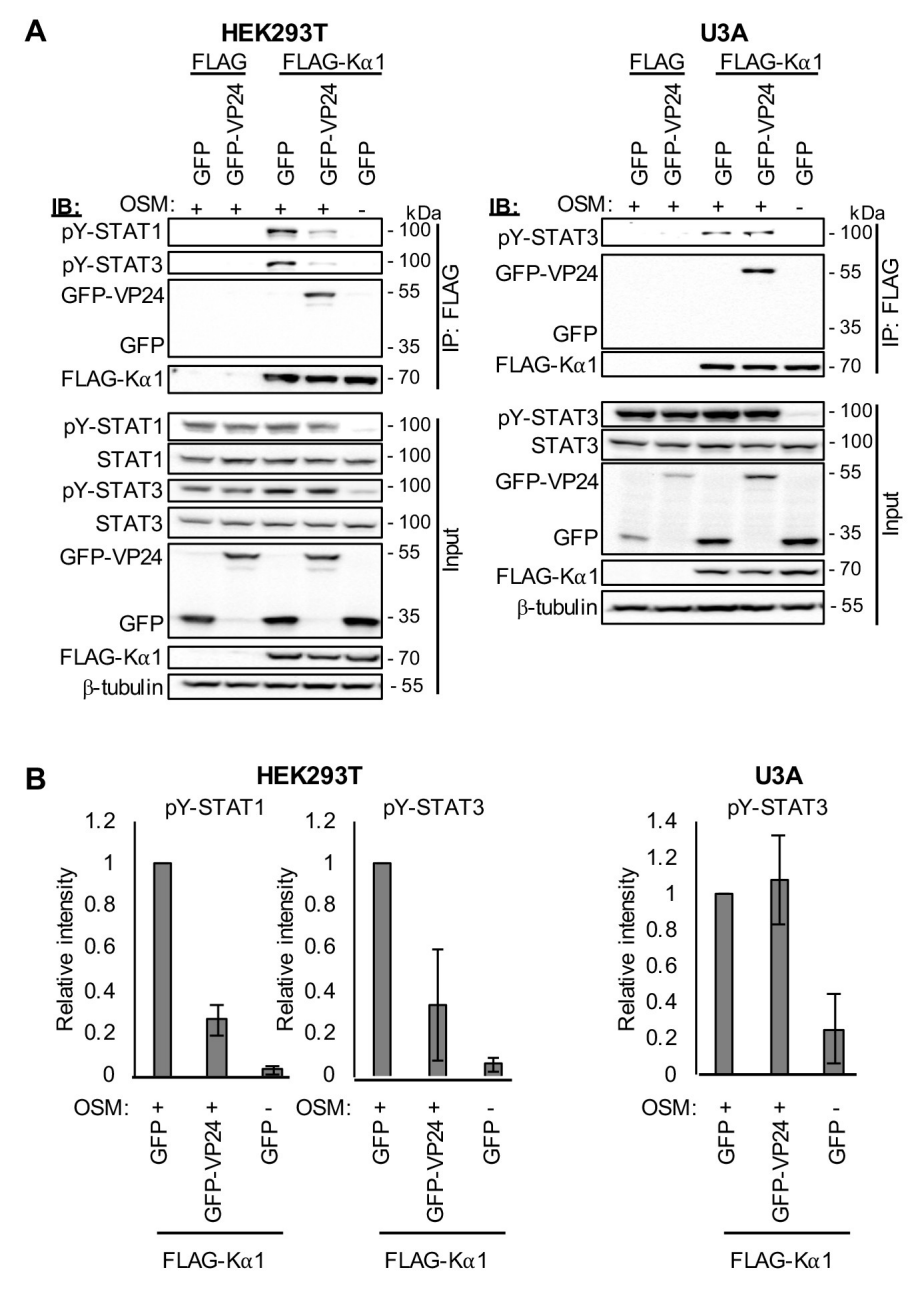
EBOV VP24 inhibits Kα1-STAT3 interaction, dependent on STAT1. (A) HEK293T or U3A cells co-transfected to express the indicated proteins were treated 24 h post-transfection with or without OSM (10 ng/ml, 15 min) before lysis and immunoprecipitation for FLAG. Lysates (input) and immunoprecipitates (IP) were analysed by IB using antibodies against the indicated proteins. Expanded images of all membranes are shown in S8 Fig. (B) Images of membranes such as those shown in (A) were analysed using Image Lab software to calculate the intensity of bands for pY-STAT1 and pY-STAT3 in IP samples; values for the different samples were calculated relative to the intensity of the corresponding FLAG-Kα1 IP band and then normalised to control samples (FLAG-Kα1/GFP treated with OSM); the histograms show mean ± SD, n ≥ 2 assays.

VP24 antagonises STAT1 responses by competitive binding to members of the NPI-1 sub-family of karyopherins [12,13,15], including Kα1 that is also reported to mediate STAT3 nuclear import [26–28]. The non-NPI-1 karyopherin Kα4 has also been implicated in STAT3 nuclear import [24,27,28], but VP24 was reported not to bind Kα4 [13], and we confirmed a lack of interaction (S5 Fig). Thus, VP24 antagonism of STAT3 would appear not to involve competitive binding to Kα4, but potentially involves competitive binding to Kα1. To examine whether VP24 expression can displace STAT3 from Kα1, we performed immunoprecipitation of FLAG-Kα1 from OSM-treated HEK293T cells (as previously used to analyse effects of VP24 on IFN-activated pY-STAT1-karyopherin interactions [12,13]) or U3A cells. Cells were co-transfected to express FLAG-Kα1 with GFP-VP24 or GFP, before OSM treatment and lysis for immunoprecipitation (Fig 5A). pY-STAT1, pY-STAT3 and GFP-VP24 co-precipitated specifically with Kα1 as expected, consistent with reports that STAT1 and STAT3 are Kα1 cargoes [8,26,27], and VP24 can interact with Kα1 [12,13]. Clearly, for both pY-STAT1 (as expected [12,13]) and pY-STAT3, the amount co-precipitated with Kα1 from HEK293T cells was reduced by VP24, consistent with competitive binding (Fig 5A and 5B). Importantly, although a number of IFN-antagonists suppress STAT phosphorylation [5], VP24 did not affect levels of pY-STAT1 or pY-STAT3 in lysates (Fig 5A), indicating that reduced interaction of Kα1 with STAT3 is not due to altered phosphorylation. Thus, it appears that VP24 can compete with STAT3-containing complexes for Kα1 interaction, similar to its effect on STAT1.

Intriguingly, however, co-immunoprecipitation assays in U3A cells indicated that VP24 does not affect Kα1-pY-STAT3 interaction (Fig 5A and 5B), despite clear impact on STAT3 responses in these cells (Fig 4). It has been suggested that karyopherin interactions of STAT homo- and heterodimers might differ [24,25]. Our data support this idea, providing evidence that the association of Kα1 with STAT3-STAT1 heterodimers differs from its association with STAT3 homodimers, as VP24 binding to Kα1 competes with the former, but not the latter interaction. The competitive nature of binding of VP24 and STAT1 to Kα1 results from the binding of both proteins at a non-classical STAT1/VP24-binding site. Our data thus suggest that STAT3-STAT1 heterodimers bind at, or proximal to, this non-classical site and so can be displaced by VP24 (Fig 5A and 5B). Since STAT1 homodimers and STAT1-STAT2 heterodimers also bind to this site [10], this might represent a common interface for STAT1-containing complexes, such that competitive binding by VP24 would be likely to occur for heterodimers activated by other cytokines/mediators (e.g. STAT4-STAT1 heterodimers). However, it would appear that STAT3 homodimers bind in a different fashion, possibly at a distinct site such as in a conventional cargo binding region, resulting in a lack of competition since VP24 binds elsewhere. Identification of the site in Kα1 that mediates binding to STAT3 homodimers, and other molecular details such as whether the binding requires additional host factors, will form the focus of future research. Nevertheless, since the data from U3A cells indicate that STAT3 homodimers bind to Kα1 *via* a site not competitively bound by VP24, it appears that an alternative mechanism is required to antagonise signalling by these complexes.

### VP24 does not inhibit STAT3 binding to DNA

**Fig 6:**
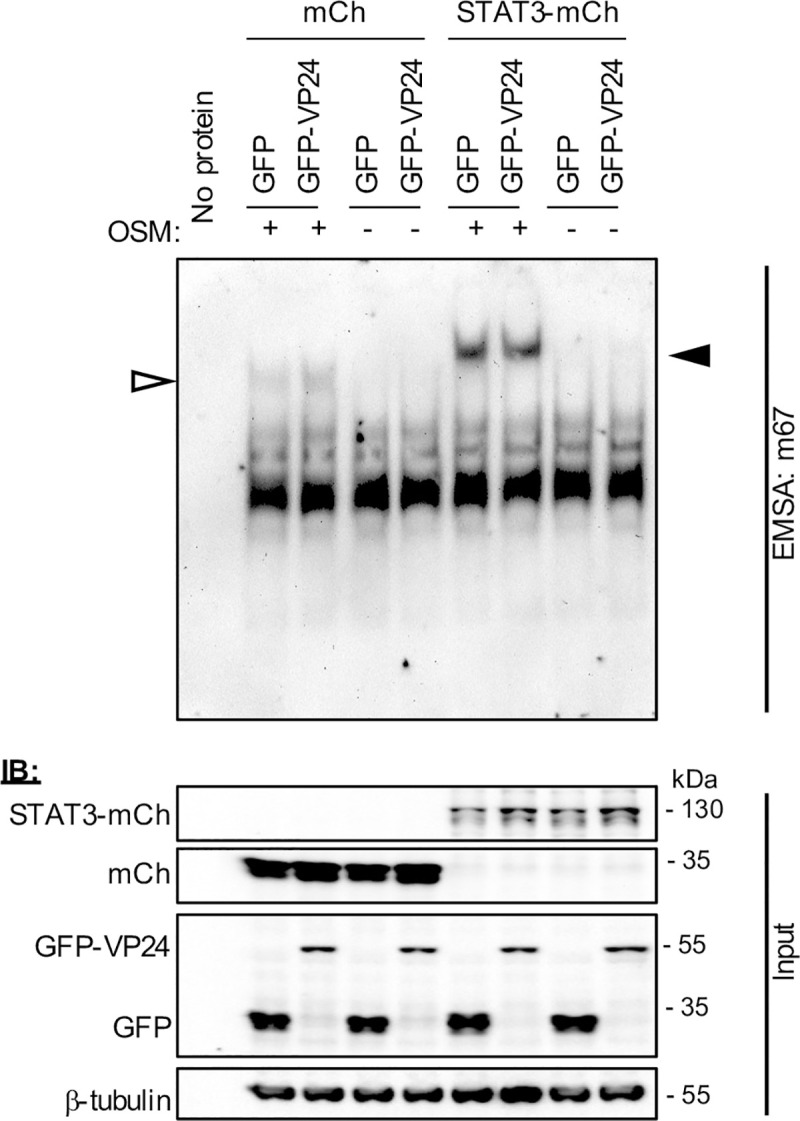
EBOV VP24 does not prevent interaction of STAT3 with target DNA. *Upper panel*: U3A cells co-transfected to express the indicated proteins were treated 24 h post-transfection with or without OSM (10 ng/ml, 15 min) before lysis and incubation of equal amounts of cell lysate protein or no lysate control (no protein) with digoxigenin-labelled m67 probe. EMSA reactions were resolved on 4.5% polyacrylamide gel in 0.5 x TBE, before transfer to a nylon membrane and IB for digoxigenin. Results are representative of 3 independent assays. Filled and unfilled arrowheads indicate bands consistent with DNA complexes with STAT3-mCherry and endogenous STAT3, respectively. *Lower panel*: Cell lysates were analysed by IB (input) using antibodies against the indicated proteins.

Reports supporting constitutive nuclear trafficking of STAT3 suggest that STAT3 accumulates in the nucleus in response to cytokine activation due to intra-nuclear interactions/sequestration, such as through induced DNA binding [25]. We therefore considered that VP24 may inhibit STAT3 nuclear accumulation in U3A cells by inhibiting the capacity of STAT3 to bind to DNA, similar to the antagonistic mechanism of RABV P-protein for STAT1, where the P-protein binds proximal to or within the STAT1 DNA binding domain [44,45]. To assess DNA binding by STAT3, we performed electrophoretic mobility shift assay (EMSA) analysis of cell lysates using the m67 probe (Fig 6), which is a high affinity variant of the sis-inducible element from the *c-fos* gene, commonly used to analyse STAT3-DNA binding [46–48]. OSM induced clear DNA binding of both endogenous and over-expressed STAT3 in the absence and presence of VP24, with VP24 having no evident inhibitory effect. Thus, the principal mechanism of antagonism does not appear to involve a direct hindrance of STAT3-DNA interaction.

### VP24 interacts with STAT3, independently of VP24-karyopherin binding

**Fig 7:**
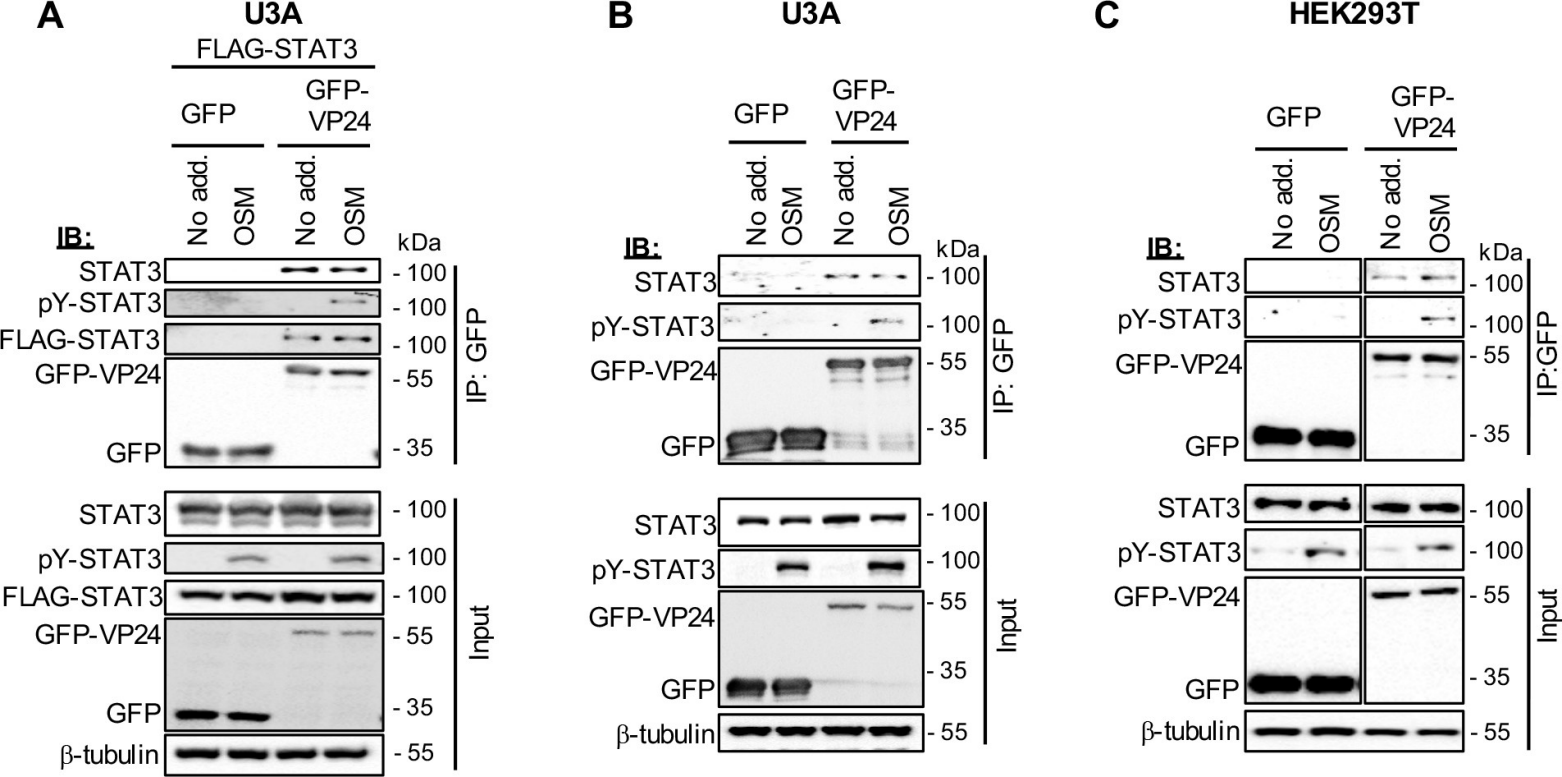
EBOV VP24 interacts with STAT3. (A) U3A cells co-transfected to express FLAG-STAT3 and GFP or GFP-VP24 as indicated were treated 24 h post-transfection with or without OSM (10 ng/ml, 30 min) before lysis, immunoprecipitation for GFP, and IB, as described in the legend to Fig 5. (B, C) U3A (B) or HEK293T (C) transfected to express the indicated proteins were treated with or without OSM (10 ng/ml, 15 min) before immunoprecipitation for GFP and IB for endogenous STAT3. Results are representative of 2 independent assays and show data from a single blot with intervening and marker lanes removed. Expanded images of all membranes are shown in S9 and S10 Figs.

While there is substantial evidence that VP24 mediates antagonism of STAT1 by competitive binding to karyopherins [12–15], recombinant purified VP24 and STAT1 proteins were reported to interact *in vitro* [49], suggesting that direct VP24-STAT1 binding may also contribute to antagonism. However, immunoprecipitation of VP24 expressed in mammalian cells and analysis of co-precipitated proteins by immunoblotting (IB) or mass spectrometry did not detect a VP24-STAT1 interaction [15,50,51], so there is currently a lack of clear evidence that this is significant to STAT1 antagonist function. Nevertheless, since many IFN-antagonists inhibit STATs through direct or indirect physical interaction [5], we tested whether VP24 can bind to STAT3. Endogenous and transfected STAT3 co-precipitated with VP24 from U3A cells (Fig 7A and 7B), and reciprocal immunoprecipitation *via* STAT3 confirmed an association (S6 Fig). Thus, VP24 can interact with STAT3 independently of STAT1, consistent with data for antagonism of OSM/STAT3 signalling (Fig 4). We also confirmed co-precipitation of STAT3 with VP24 from HEK293T and COS7 cells (Figs 7C and S10). While the association of VP24 and STAT3 is clearly specific compared with controls, the amount of STAT3 detected in immunoprecipitates of VP24 appears relatively low, potentially reflecting a transient interaction and/or poor retention of the complex during cell lysis and immunoprecipitation. Since immunoprecipitation from cells does not differentiate direct and indirect interactions, it is also possible that VP24-STAT3 interaction is mediated *via* other cellular components, which may result in some dissociation during lysis/immunoprecipitation.

pY-STAT3 co-precipitated with VP24 exclusively from OSM-treated cells as expected since pY-STAT3 is induced by OSM. However, IB for total STAT3 (detecting both non-phosphorylated and phosphorylated forms), indicated comparable co-precipitation with VP24 from cells treated with or without OSM (Figs 7 and S10). Thus, VP24 appears to interact with both the phosphorylated and non-phosphorylated STAT3. To confirm this, we examined the association of VP24 with STAT3-R609Q/Y705F, which contains mutations at the pY site (Y705F) and SH2 domain (R609Q), preventing cytokine-induced phosphorylation and dimerisation [52]. FLAG-tagged STAT3-R609Q/Y705F and FLAG-tagged wild-type (WT) STAT3 co-precipitated with VP24 to a similar extent (S7 Fig), confirming that VP24-STAT3 interaction is independent of STAT3 activation.

**Fig 8:**
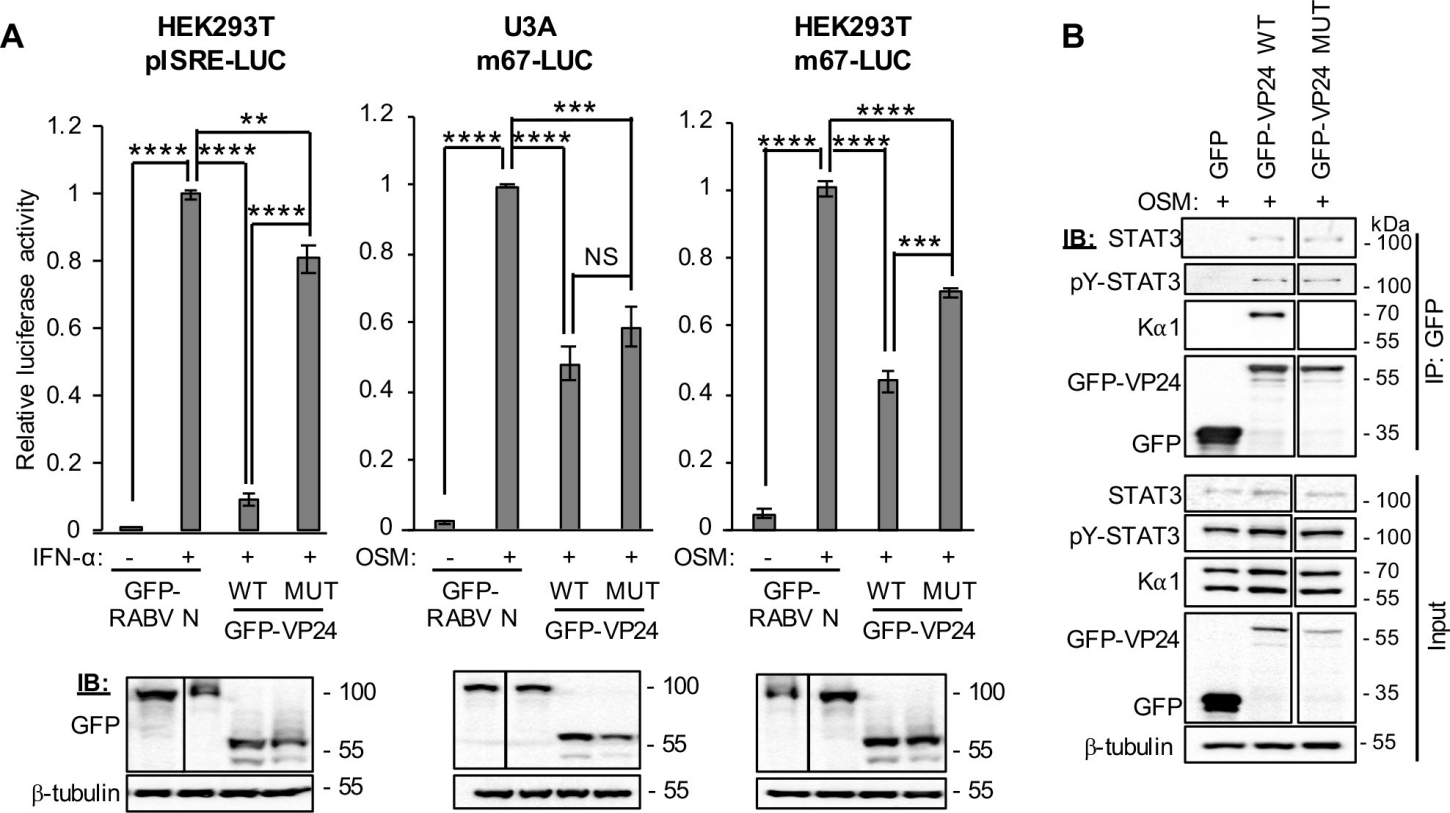
Antagonism of STAT3 by EBOV VP24 in U3A cells is independent of VP24-karyopherin interaction. (A) *upper panel*: HEK293T or U3A cells co-transfected with pISRE-LUC or m67-LUC plasmid, pRL-TK plasmid, and plasmids to express the indicated proteins, were treated 8 h (IFN-α) or 16 h (OSM) post-transfection with or without IFN-α (1,000 U/ml for 16 hours) or OSM (10 ng/ml for 8 h) before determination of relative luciferase activity (mean ± SEM; n ≥ 3 independent assays); *lower panel*: cell lysates used in representative assays were analysed by IB for GFP and β-tubulin. Statistical analysis used Student’s *t*-test; **, p < 0.01; ***, p < 0.001; ****, p < 0.0001; NS, not significant. (B) U3A cells transfected to express the indicated proteins were treated with OSM before immunoprecipitation for GFP and IB, as described in the legend to Fig 7. Results are representative of 2 independent assays and show data from a single blot with intervening and marker lanes removed. Expanded images of membranes are shown in S11 Fig.

To further investigate the antagonistic mechanism, we analysed a karyopherin-binding deficient VP24 protein (VP24 MUT), wherein mutations of key residues at the VP24:karyopherin interface (L201A/E203A/P204A/D205A/S207A) strongly impair karyopherin binding and STAT1/IFN antagonism [15]. The effect of the mutations in inhibiting STAT1-antagonist function was confirmed using a STAT1/2-IFN-dependent luciferase reporter assay (using pISRE-LUC plasmid), which indicated an almost nine-fold increase in luciferase activity in IFN-α-treated HEK293T cells expressing mutated protein compared with WT protein (Fig 8A; left panel). Analysis using the STAT3/OSM-dependent signalling assay (using m67-LUC plasmid) in U3A cells showed no significant impact of the mutations on VP24 inhibitory activity (Fig 8A; middle panel), indicating that specific antagonism of STAT3 by VP24 is independent of karyopherin-binding activity, consistent with the lack of an effect of VP24 on Kα1-STAT3 association in U3A cells. Assays of STAT3/OSM-dependent signalling in HEK293T cells, however, indicated some dependence on karyopherin-binding, probably reflecting a contribution to signalling by STAT3-STAT1 heterodimers (Fig 8A; right panel), consistent with the capacity of VP24 to compete with STAT1 and STAT3 for Kα1 binding in these cells. Thus, it appears that, in contrast to STAT1 (and STAT3-STAT1 heterodimers), antagonism of signalling by STAT3 homodimers is independent of VP24-karyopherin binding. Consistent with this, the mutations had no evident effect on VP24-STAT3 interaction in U3A cells (Fig 8B). Taken together, these data indicate that the nuclear trafficking mechanisms of STAT1 and STAT3 are distinct, and, accordingly, antagonism by VP24 uses different mechanisms, likely including competition with STAT1-containing complexes for karyopherin binding, as well as physical interaction with STAT3, which effects cytoplasmic localisation. This is perhaps consistent with reports that STAT3 nuclear import can be mediated by multiple karyopherins including non-NPI-1 karyopherins, such as Kα4 [24,27,28], which may have necessitated the development in VP24 of a distinct, karyopherin-independent strategy to antagonise STAT3.

Taken together, our data indicate that VP24 can inhibit STAT3 signalling by two distinct mechanisms, consistent with important roles for global shutdown of STAT3 in EBOV infection. Since OSM stimulation of cells can impair viral replication, it appears that STAT3 antagonism forms part of the viral strategy to counteract antiviral processes in the infected cell. However, given the established roles for STAT3 in regulating the broader immune response and other diverse cellular and physiological processes, it is possible that STAT3 antagonism is also related to pathogenic processes such as the dysregulation of inflammation, coagulation and mucosal wound healing, observed during EBOV infection [6,21–23,53,54]. Recent reports indicate that STAT3 antagonism by MUV is associated with neurovirulence *in vivo* [55], and suppression of IL-6 signalling by influenza A virus early in infection contributes to a cytokine storm implicated in disease severity [56]. Interestingly, although the IFN-antagonist VP40 of the filovirus Marburg virus does not specifically target STATs, it inhibits upstream kinases resulting in inhibition of activation of both STAT1 and STAT3 [57]. Together these data indicate that potent suppression of STAT3 responses by filoviruses may contribute to excessive inflammatory responses associated with severe haemorrhagic fever. The apparent importance of STAT3 targeting to filoviruses, and previous reports of roles in infection by paramyxoviruses and rhabdoviruses, also indicates that specific and direct antagonism of STAT3 is important to diverse pathogens in the order *Mononegavirales* [16–20]. These data suggest that virus-STAT3 interactions could provide new directions for the development of antivirals for diverse pathogens.

Beyond the implications for viral infection, the study also provides, to our knowledge, the first clear indication of distinct nuclear import strategies for STAT3 homodimers and heterodimers, potentially accounting for the contrasting trafficking models previously proposed [24–28]. Together with our recent finding that lyssaviruses differentially target STAT3 dimers to modulate cytokine-induced transcription [20], this supports the idea that these complexes have distinct roles in signalling by STAT3, a pleiotropic molecule important to processes including cancer, development and immunity.

## Materials and methods

### Plasmids and cell culture

Constructs to express EBOV-VP24 and MUV-V fused to GFP were generated by PCR amplification from pCAGGS-FLAG-VP24 (kindly provided by Christopher Basler, Georgia State University) and MUV V-FLAG (a gift from Curt Horvath [16], Addgene plasmid #44908), and cloning into the pEGFP-C1 vector C-terminal to GFP (Clontech). Constructs to express mCherry- or FLAG-tagged STAT3-WT were kind gifts from Marie Bogoyevitch (University of Melbourne), and the constructs to express FLAG-tagged Kα1 and Kα4 were a kind gift from Christopher Basler (Georgia State University). Primers/constructs to express FLAG-tagged STAT3-R609Q/Y705F were a kind gift from Daniel Gough (Hudson Institute of Medical Research). Other constructs have been described elsewhere [18,43,58]. U3A (a kind gift from George Stark, Lerner Research Institute, Cleveland Clinic), COS7, E6 Vero, HeLa and HEK293T cells were maintained in DMEM supplemented with 10% FCS and GlutaMAX (Life Technologies), 5% CO_2_, 37°C. Transfections used Lipofectamine 2000 (Invitrogen), Lipofectamine 3000 (Invitrogen), or FuGene HD (Promega), according to the manufacturer’s instructions.

### Virus infection

All work with infectious virus was conducted at Physical Containment Level 4 (PC4) at the Australian Centre for Disease Preparedness (ACDP, formerly AAHL). EBOV infections used Mayinga 1976 isolate, which was originally received from NIH Rocky Mountain Laboratories and passaged three times in Vero cells at ACDP after receipt.

### CLSM

For analysis of STAT3 localisation, cells growing on coverslips transfected with plasmids or infected with EBOV (MOI = 10) were incubated in serum-free-(SF)-DMEM for 1 h and treated without or with 10 ng/ml recombinant human OSM (BioVision) for 15 min (analysis of fixed/immunostained cells) or 30 min (analysis of STAT3-mCherry in living cells) before fixation using 3.7% formaldehyde (10 min, room temperature (RT) for transfected cells) or 4% paraformaldehyde (48 h, 4°C for infected cells), followed by 90% methanol (5 min, RT) and immunostaining. Antibodies used for were: anti-STAT3 (Santa Cruz, sc-482; or Cell Signaling Technology, 9139), monoclonal (Absolute Antibody, Ab00692-23.0) or polyclonal (rabbit clone #691, final bleed 1410069) anti-EBOV NP, and anti-mouse or anti-rabbit Alexa Fluor 488, 568 or 647 secondary antibodies (ThermoFisher Scientific). Imaging used a Leica SP5 or Nikon C1 inverted confocal microscope with 63 X objective. For live cell analysis, cells were imaged in phenol-free DMEM using a heated chamber. Digitised confocal images were processed using Fiji software (NIH). To quantify nucleocytoplasmic localisation, the ratio of nuclear to cytoplasmic fluorescence, corrected for background fluorescence (Fn/c), was calculated for individual cells expressing transfected protein [18,20,43]; mean Fn/c was calculated for n ≥ 24 cells for each condition in each assay.

### Analysis of the effect of OSM on EBOV replication

E6 Vero cells were treated without or with 10 ng/ml or 20 ng/ml OSM for 24 h before removal of media and infection with EBOV (MOI = 1, 1 h). The inoculum was then replaced with media without or with OSM to continue the treatment for a further 24 h, 48 h or 72 h post-infection before collection of supernatant. Alternatively, E6 Vero cells were infected with EBOV (MOI = 1, 1 h) before replacement of inoculum with media for 24 h. Cells were then treated without or with OSM (10 ng/ml or 20 ng/ml) before collection of supernatant at 24 h, 48 h and 72 h post-treatment. For quantification of virus in samples, RNA was extracted using the MagMAX-96 Viral RNA Isolation Kit (Applied Biosystems) utilizing the Kingfisher Flex (ThermoFisher Scientific) before RT-qPCR (AgPath-ID One-step reverse transcription-PCR kit, Applied Biosystems) targeting the NP gene [35]. Copy numbers were calculated using a standard curve. Assays were performed with biological triplicates.

### Luciferase reporter gene assays

Cells were co-transfected with m67-LUC or pISRE-LUC (in which Firefly luciferase expression is under the control of a STAT3 or STAT1/2-dependent promoter, respectively) and pRL-TK (transfection control, from which *Renilla* luciferase is constitutively expressed), as previously described [18,59], together with protein expression constructs. Cells were treated 16 h (OSM) or 8 h (IFN-α) post-transfection with or without OSM (10 ng/ml for 8 h) or IFN-α (1,000 U/ml for 16 hours) before lysis using Passive Lysis Buffer (Promega). Firefly and *Renilla* luciferase activity was then determined in a dual luciferase assay using a BMG CLARIOstar plate reader, as previously described [18,59]. Briefly, the ratio of Firefly to *Renilla* luminescence was determined for each condition, and then calculated relative to that determined for GFP-N-protein-expressing cells treated with OSM (relative luciferase activity). Data from ≥ 3 independent assays were combined, where each assay result is the mean of three replicate samples.

### Co-immunoprecipitation

Transfected cells were incubated in SF-DMEM (3 h) before treatment with or without OSM (10 ng/ml, 15 min for endogenous STAT3 or 30 min for transfected STAT3), lysis and immunoprecipitation using GFP-Trap or RFP-Trap Agarose beads (Chromotek) or Anti-FLAG M2 Magnetic beads (Sigma-Aldrich), according to the manufacturer’s instructions. Lysis and wash buffers were supplemented with PhosSTOP (Roche), cOmplete Protease Inhibitor Cocktail (Roche) and 10 mM NaF. Lysates and immunoprecipitates were analysed by SDS-PAGE and IB using antibodies against STAT3 (above), pY-STAT3 (Cell Signaling Technology, 9145), STAT1 (Cell Signaling Technology, 14994), pY-STAT1 (Tyr701, Cell Signaling Technology, 9167), FLAG (Sigma-Aldrich, F1804), GFP (Roche Applied Science, 11814460001), mCherry (Abcam, ab167453), Kα1 (Abcam, ab154399) and β-tubulin (Sigma-Aldrich, T8328), and HRP-conjugated secondary antibodies (Merck). Visualisation of bands used Western Lightning chemiluminescence reagents (PerkinElmer). Densitometric analysis was performed using Image Lab (Bio-Rad) software.

### EMSA

Transfected cells were incubated in SF-DMEM (2 h) before treatment with or without OSM (10 ng/ml, 15 min) and lysis in EMSA lysis buffer (20 mM Hepes (pH 7.0), 300 mM NaCl, 20% (v/v) glycerol, 10 mM KCl, 1 mM MgCl_2_, 0.5 mM DTT, 0.1% (v/v) Triton X-100) as previously described [60], except that lysis buffer was supplemented with PhosSTOP (Roche), cOmplete Protease Inhibitor Cocktail (Roche) and 10 mM NaF. 10 ng of clarified cell lysate (calculated using Pierce Microplate BCA Protein Assay Kit—Reducing Agent Compatible, ThermoFisher Scientific) was incubated with 1 ng of digoxigenin-labelled m67 probe (double-stranded; 5’-AGCTTCATTTCCCGTAAATCCCTA-3’) for 15 min at RT in a 20 μl binding reaction containing 4 μl binding buffer (DIG Gel Shift Kit, Roche), 1 μg poly[d(I-C)] and 0.1 μg poly-Lysine [61]. DNA-protein complexes were resolved on a 4.5% polyacrylamide gel in 0.5 x TBE running buffer (4°C), before electrophoretic transfer to a nylon membrane and IB using anti-Digoxigenin-AP Fab fragments (Roche). Visualisation of bands used CDP-Star chemiluminescence reagents (Roche).

### RT-qPCR analysis of socs3 expression

Transfected HEK293T cells were incubated in SF-DMEM (3 h) before treatment without or with OSM (10 ng/ml, 45 min) and RNA extraction (ReliaPrep RNA Cell Miniprep System, Promega). cDNA was generated using oligo(dT)_20_ primer (GoScript Reverse Transcription System, Promega), before qPCR using primers for *socs3* and *gapdh*, and iTaq Universal SYBR Green Supermix (Bio-Rad). *Socs3* expression was normalised to *gapdh* using the comparative C_T_ method [59,62], and then calculated relative to that for control (GFP or GFP-RABV N)-expressing cells treated with OSM. Data from 4 independent assays were combined, where the value from each assay is the mean of technical replicates. Primer sequences were: 5’-GGAGTTCCTGGACCAGTACG-3’ and 5’-TTCTTGTGCTTGTGCCATGT-3’ for *socs3*; 5’-GAAGGTGAAGGTCGGAGTC-3’ and 5’-GGTCATGAGTCCTTCCACGAT-3’ for *gapdh*.

### Statistical analysis

Unpaired two-tailed Student’s *t*-test was performed using Prism software (version 7, GraphPad).

## Supporting information

S1 Fig EBOV forms NP enriched inclusions in infected cells. (A) COS7 (left panel) or E6 Vero (right panel) cells infected with EBOV (MOI 10) were treated 72 h post-infection with or without OSM (10 ng/ml, 15 min) before fixation, immunofluorescent staining for EBOV NP (polyclonal antibody, rabbit clone #691, final bleed 1410069, red) and STAT3 (green), and analysis by CLSM. DAPI (blue) was used to localise nuclei. Images are representative of ≥ 5 fields of view for each condition. Arrowheads indicate accumulation of NP in discrete cytoplasmic regions/inclusions. Scale bars, 30 μm. (B) Images such as those shown in A were analysed to calculate the Fn/c for STAT3 (mean ± SEM, n ≥ 24 cells for each condition). Statistical analysis used Student’s *t*-test; ****, p < 0.0001.
(TIF)

S2 Fig EBOV VP24 antagonises STAT3 in HeLa and Vero cells. HeLa (upper panel) or Vero (lower panel) cells transfected to express the indicated proteins were treated 24 h post-transfection with or without OSM (10 ng/ml, 15 min) before fixation, immunofluorescent staining for STAT3 (red), and CLSM analysis (A) to determine the Fn/c for STAT3 (B; mean ± SEM, n ≥ 34 cells for each condition). Filled and unfilled arrowheads indicate cells with or without, respectively, detectable expression of the transfected protein. Scale bars, 30 μm. Statistical analysis used Student’s *t*-test; ****, p < 0.0001.
(TIF)

S3 Fig VP24-mediated inhibition of STAT3 is independent of NP. COS7 (upper panel) or U3A (lower panel) cells transfected to express the indicated proteins were treated 24 h post-transfection with or without OSM (10 ng/ml, 15 min) before fixation, immunofluorescent staining for STAT3 (red) and EBOV NP (blue), and CLSM analysis (A) to determine the Fn/c for STAT3 (B; mean ± SEM, n ≥ 27 cells for each condition). Arrowheads indicate cells with detectable expression of the transfected proteins. Scale bars, 30 μm. Statistical analysis used Student’s *t*-test; ****, p < 0.0001; NS, not significant.
(TIF)

S4 Fig EBOV VP24 inhibits STAT3-dependent gene expression. *Upper panel*: HEK293T cells transfected to express the indicated proteins were treated 24 h post-transfection with or without OSM (10 ng/ml, 45 min) before analysis by RT-qPCR. Histogram shows expression of *socs3* calculated relative to *gapdh* and normalised to control cells treated with OSM (mean ± SEM; n = 4 independent assays). Statistical analysis used Student’s *t*-test; *, p < 0.05; **, p < 0.01; ****, p < 0.0001. *Lower panel*: cell lysates used in a representative assay were analysed by IB for GFP and β-tubulin.
(TIF)

S5 Fig EBOV VP24 interacts with Kα1 but not Kα4. HEK293T cells co-transfected to express the indicated proteins were lysed 24 h post-transfection before immunoprecipitation for FLAG, and analysis by IB, as described in the legend to Fig 5A. Arrowheads indicate specific protein bands.
(TIF)

S6 Fig Reciprocal immunoprecipitation of EBOV VP24 with STAT3. U3A cells co-transfected to express STAT3-mCherry or mCherry and FLAG-VP24 or FLAG were treated with or without OSM before immunoprecipitation for mCherry and analysis by IB, as described in the legend to Fig 7A. Arrowheads indicate specific protein bands.
(TIF)

S7 Fig Mutation of STAT3 residues R609 and Y705 does not affect association with EBOV VP24. U3A cells co-transfected to express FLAG-STAT3 WT or R609Q/Y705F and GFP or GFP-VP24 were treated with or without OSM before immunoprecipitation for GFP, and analysis by IB, as described in the legend to Fig 7A. Arrowheads indicate specific protein bands.
(TIF)

S8 Fig Expanded images of western blots shown in Fig 5A. Full images of membranes shown in Fig 5A; arrowheads indicate specific proteins bands.
(TIF)

S9 Fig Expanded images of western blots shown in Fig 7A. Full images of membranes shown in Fig 7A; arrowheads indicate specific proteins bands.
(TIF)

S10 Fig Expanded images of western blots shown in Fig 7B and 7C and western analysis of immunoprecipitation assay using COS7 cells. (A, B) Full images of membranes shown in Fig 7B (A) and 7C (B). (C) Results of immunoprecipitation assay using COS7 cells transfected and treated as described for U3A and HEK293T cells in Fig 7B and 7C. Results are representative of 2 independent assays and show data from a single blot with intervening and marker lanes removed. Arrowheads indicate specific proteins bands.
(TIF)

S11 Fig Expanded images of western blots shown in Fig 8B. Full images of membranes shown in Fig 8B; arrowheads indicate specific proteins bands.
(TIF)

S1 Data Excel spreadsheet containing, in separate sheets, the underlying numerical data for Fig panels 1B, 2A, 2B, 3B, 4B, 4D, 5B, 8A, S1B, S2B, S3B and S4. (XLSX)
