# Supplementary figures and images for: Antagonism of STAT3 signalling by Ebola virus
Source: PLoS Pathog. 2021 Jun 24;17(6):e1009636. doi: 10.1371/journal.ppat.1009636 (PMC8224886; doi:10.1371/journal.ppat.1009636)

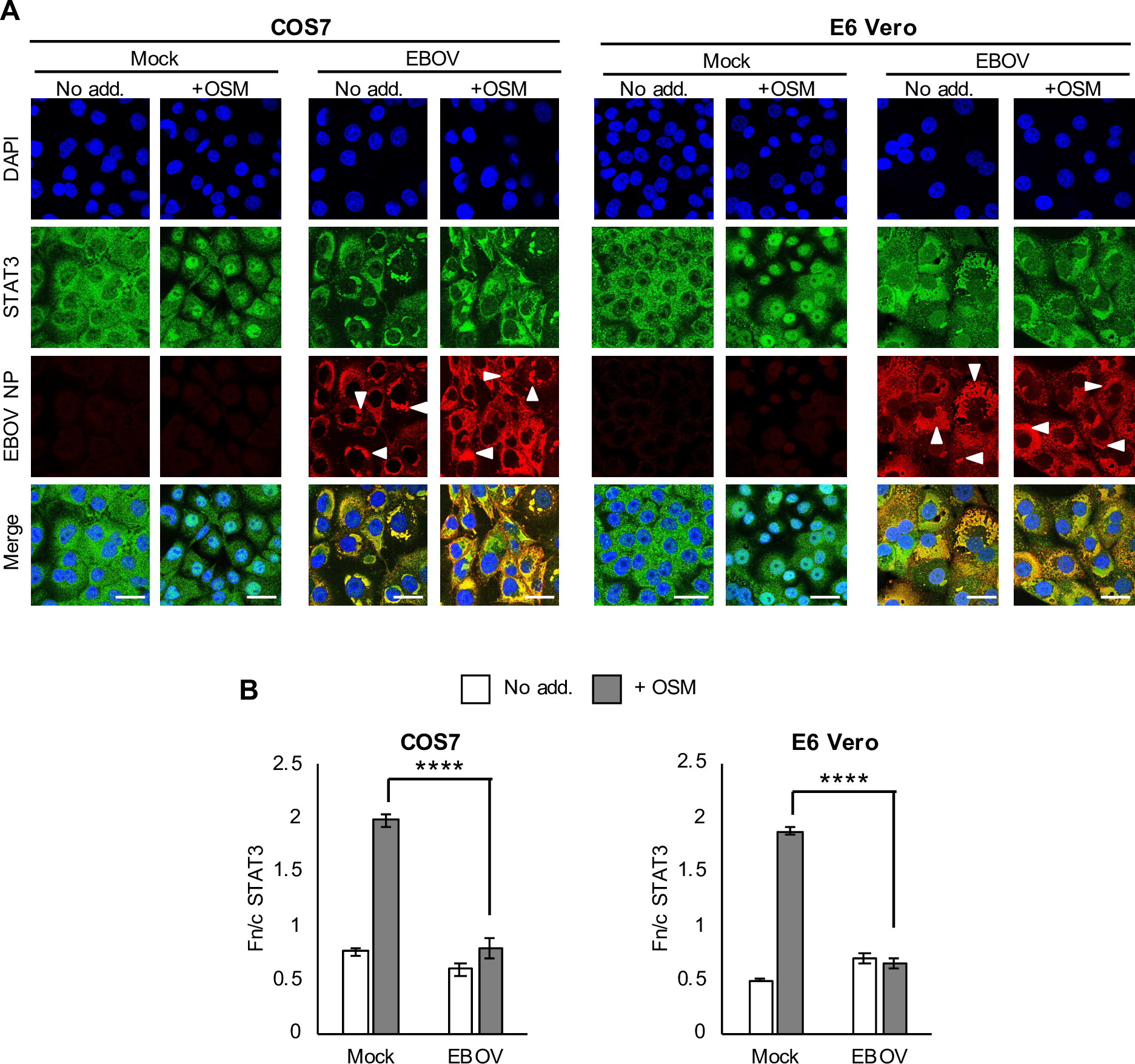

Supplement: S1 Fig — (A) COS7 (left panel) or E6 Vero (right panel) cells infected with EBOV (MOI 10) were treated 72 h post-infection with or without OSM (10 ng/ml, 15 min) before fixation, immunofluorescent staining for EBOV NP (polyclonal antibody, rabbit clone #691, final bleed 1410069, red) and STAT3 (green), and analysis by CLSM. DAPI (blue) was used to localise nuclei. Images are representative of ≥ 5 fields of view for each condition. Arrowheads indicate accumulation of NP in discrete cytoplasmic regions/inclusions. Scale bars, 30 μm. (B) Images such as those shown in A were analysed to calculate the Fn/c for STAT3 (mean ± SEM, n ≥ 24 cells for each condition). Statistical analysis used Student’s t-test; ****, p < 0.0001. (TIF) [file ppat.1009636.s001.tif]

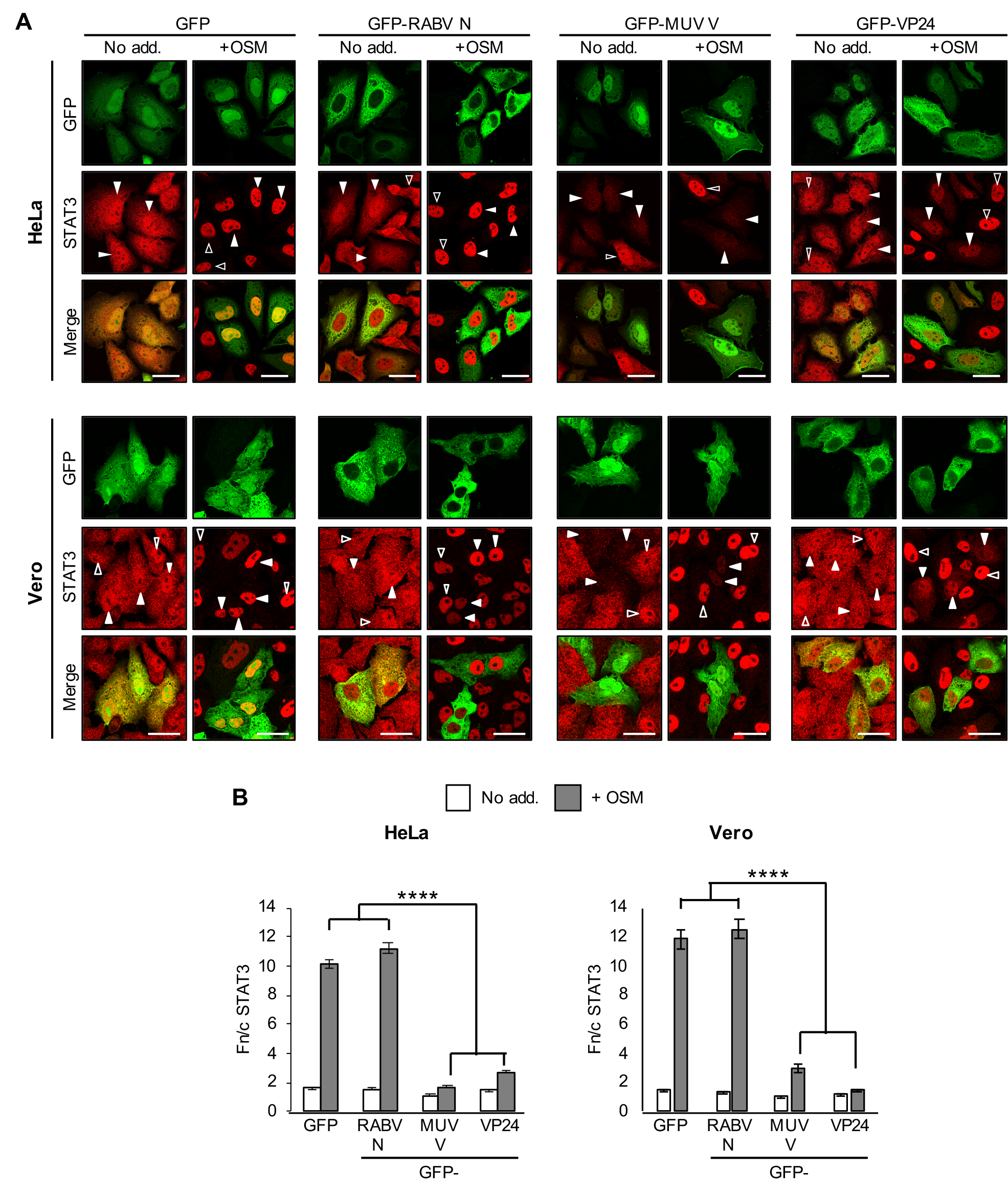

Supplement: S2 Fig — HeLa (upper panel) or Vero (lower panel) cells transfected to express the indicated proteins were treated 24 h post-transfection with or without OSM (10 ng/ml, 15 min) before fixation, immunofluorescent staining for STAT3 (red), and CLSM analysis (A) to determine the Fn/c for STAT3 (B; mean ± SEM, n ≥ 34 cells for each condition). Filled and unfilled arrowheads indicate cells with or without, respectively, detectable expression of the transfected protein. Scale bars, 30 μm. Statistical analysis used Student’s t-test; ****, p < 0.0001. (TIF) [file ppat.1009636.s002.tif]

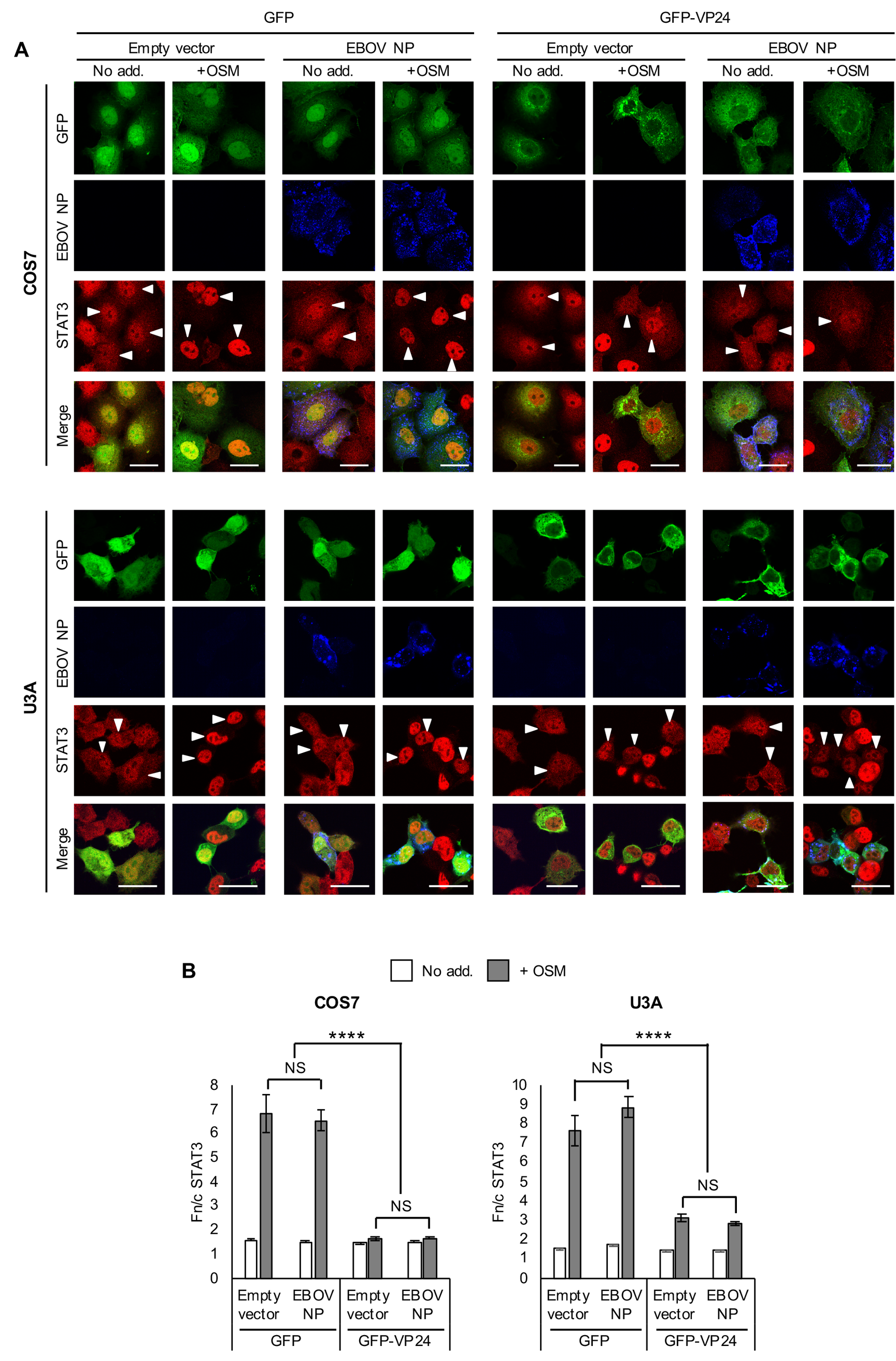

Supplement: S3 Fig — COS7 (upper panel) or U3A (lower panel) cells transfected to express the indicated proteins were treated 24 h post-transfection with or without OSM (10 ng/ml, 15 min) before fixation, immunofluorescent staining for STAT3 (red) and EBOV NP (blue), and CLSM analysis (A) to determine the Fn/c for STAT3 (B; mean ± SEM, n ≥ 27 cells for each condition). Arrowheads indicate cells with detectable expression of the transfected proteins. Scale bars, 30 μm. Statistical analysis used Student’s t-test; ****, p < 0.0001; NS, not significant. (TIF) [file ppat.1009636.s003.tif]

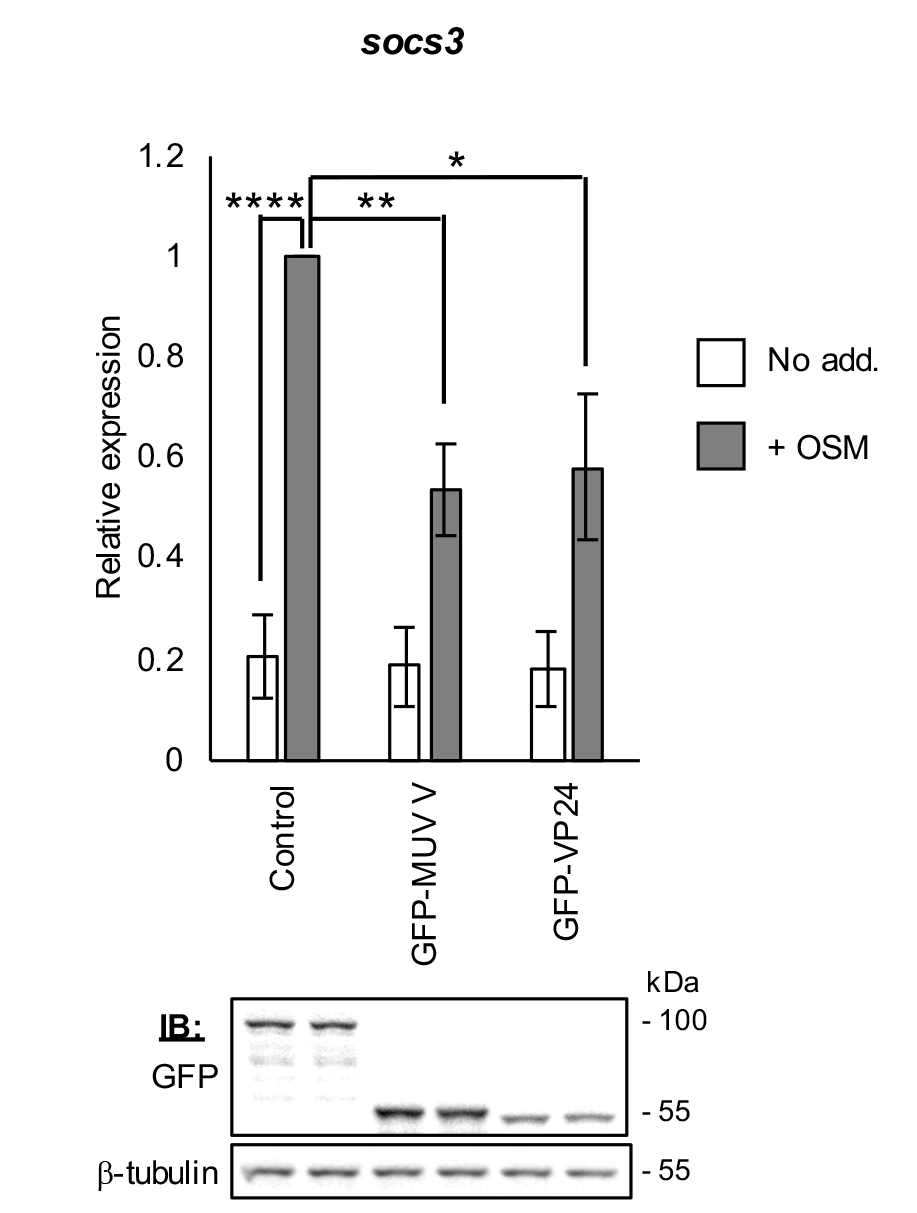

Supplement: S4 Fig — Upper panel: HEK293T cells transfected to express the indicated proteins were treated 24 h post-transfection with or without OSM (10 ng/ml, 45 min) before analysis by RT-qPCR. Histogram shows expression of socs3 calculated relative to gapdh and normalised to control cells treated with OSM (mean ± SEM; n = 4 independent assays). Statistical analysis used Student’s t-test; *, p < 0.05; **, p < 0.01; ****, p < 0.0001. Lower panel: cell lysates used in a representative assay were analysed by IB for GFP and β-tubulin. (TIF) [file ppat.1009636.s004.tif]

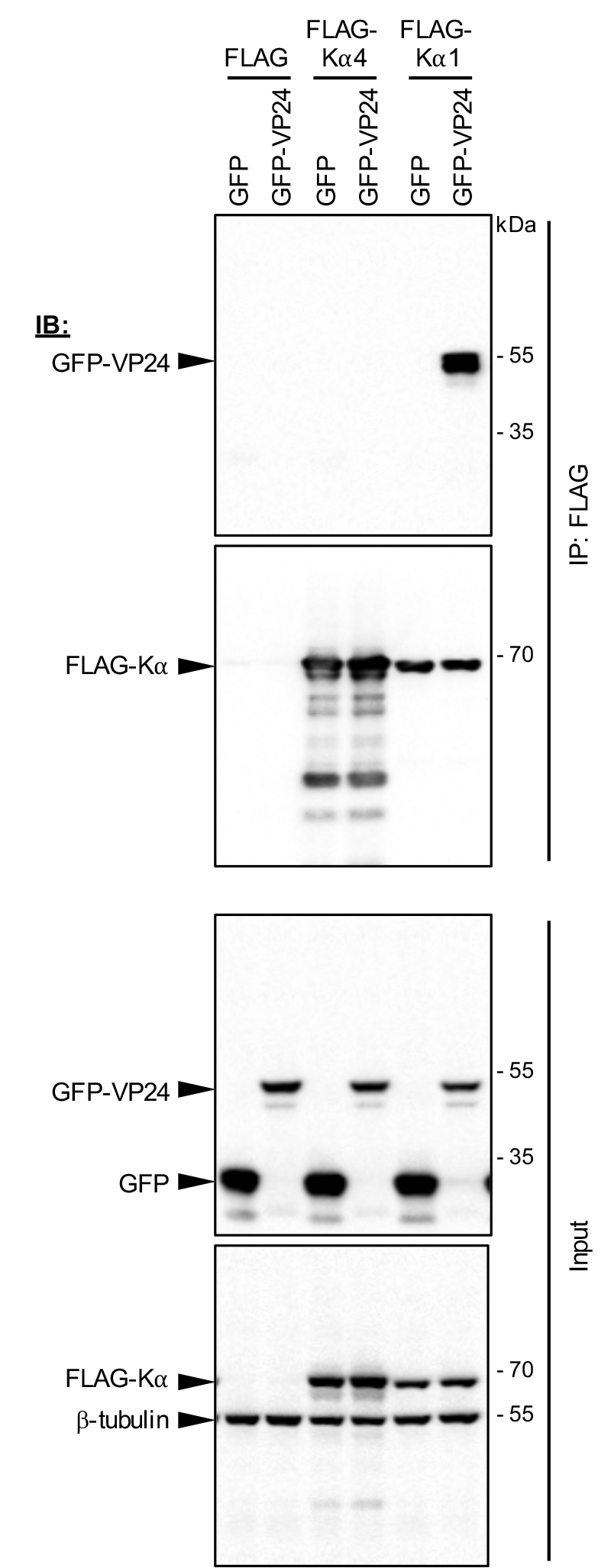

Supplement: S5 Fig — HEK293T cells co-transfected to express the indicated proteins were lysed 24 h post-transfection before immunoprecipitation for FLAG, and analysis by IB, as described in the legend to Fig 5A. Arrowheads indicate specific protein bands. (TIF) [file ppat.1009636.s005.tif]

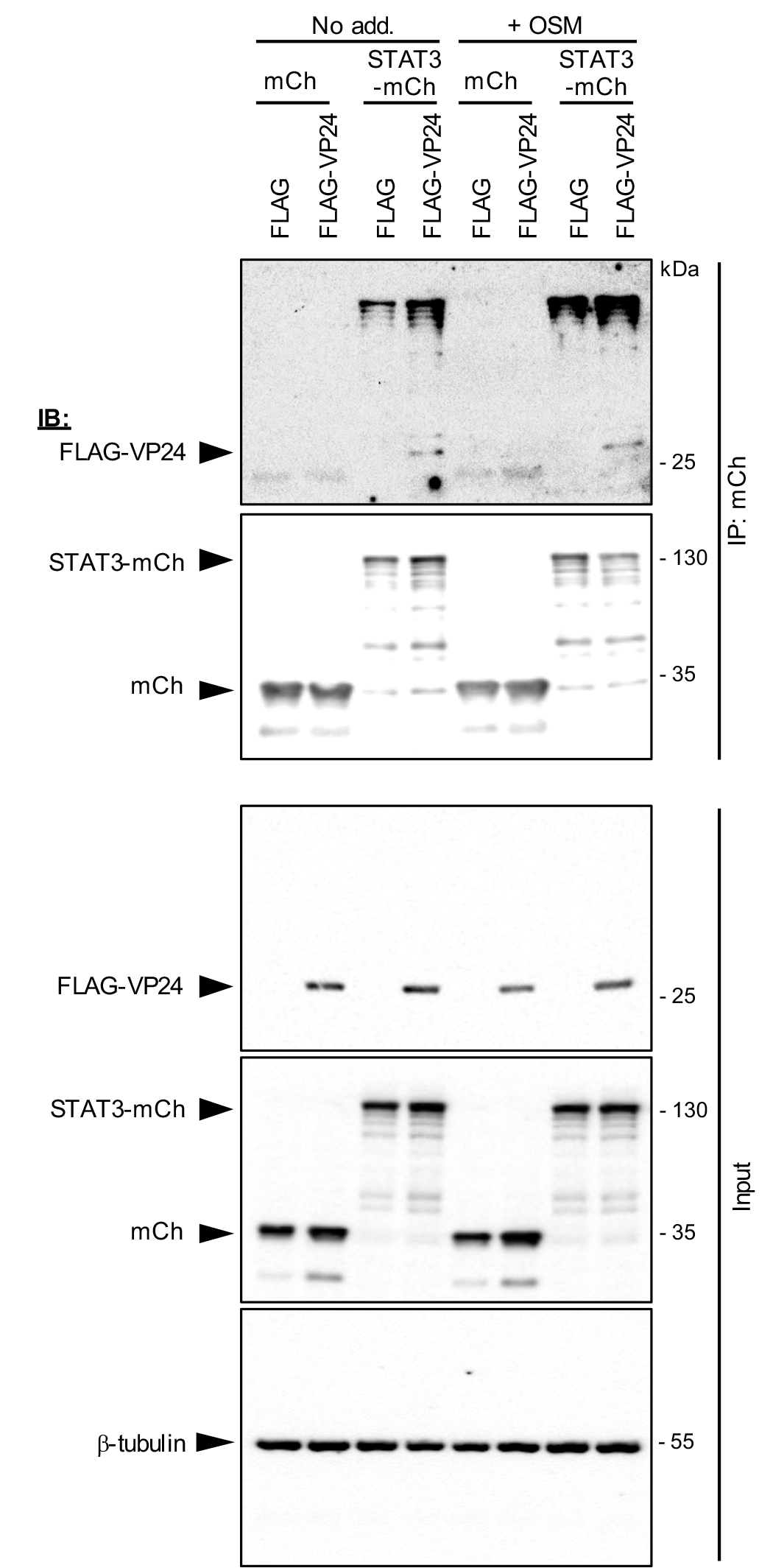

Supplement: S6 Fig — U3A cells co-transfected to express STAT3-mCherry or mCherry and FLAG-VP24 or FLAG were treated with or without OSM before immunoprecipitation for mCherry and analysis by IB, as described in the legend to Fig 7A. Arrowheads indicate specific protein bands. (TIF) [file ppat.1009636.s006.tif]

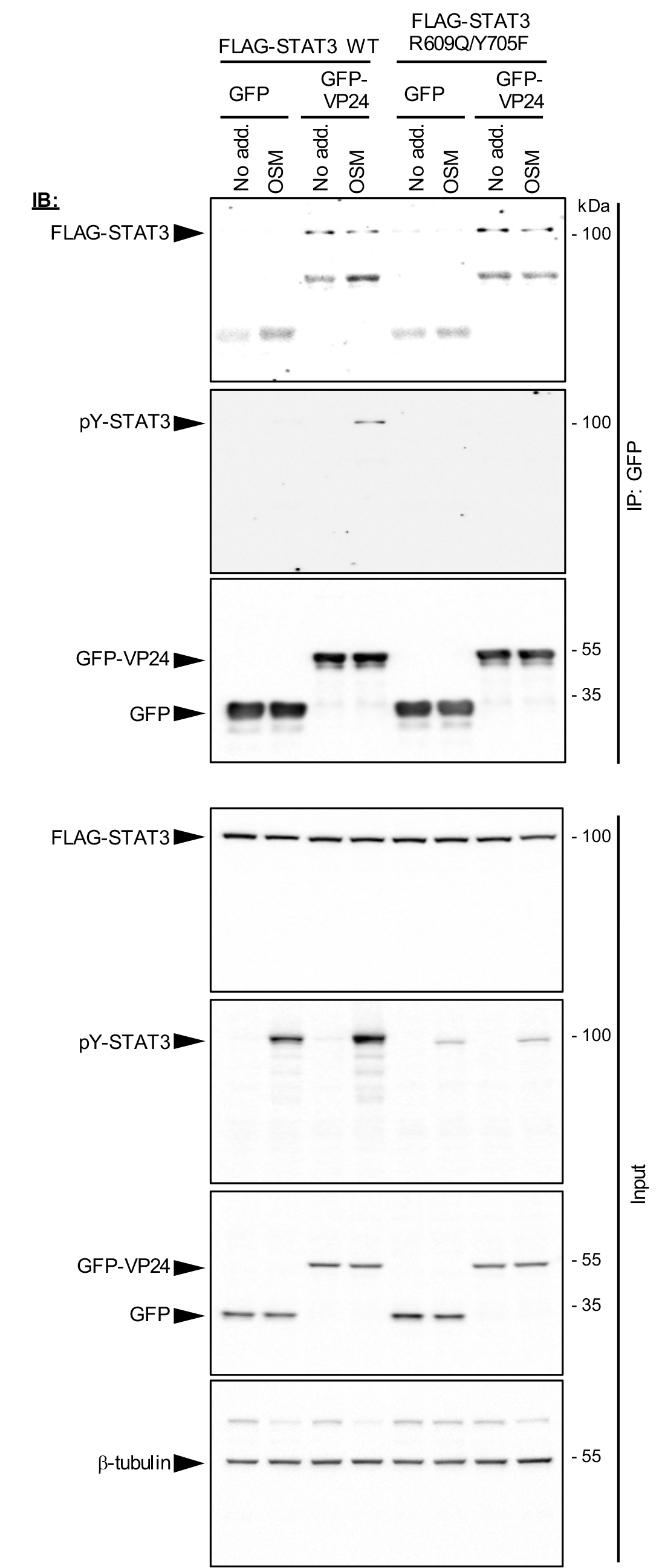

Supplement: S7 Fig — U3A cells co-transfected to express FLAG-STAT3 WT or R609Q/Y705F and GFP or GFP-VP24 were treated with or without OSM before immunoprecipitation for GFP, and analysis by IB, as described in the legend to Fig 7A. Arrowheads indicate specific protein bands. (TIF) [file ppat.1009636.s007.tif]

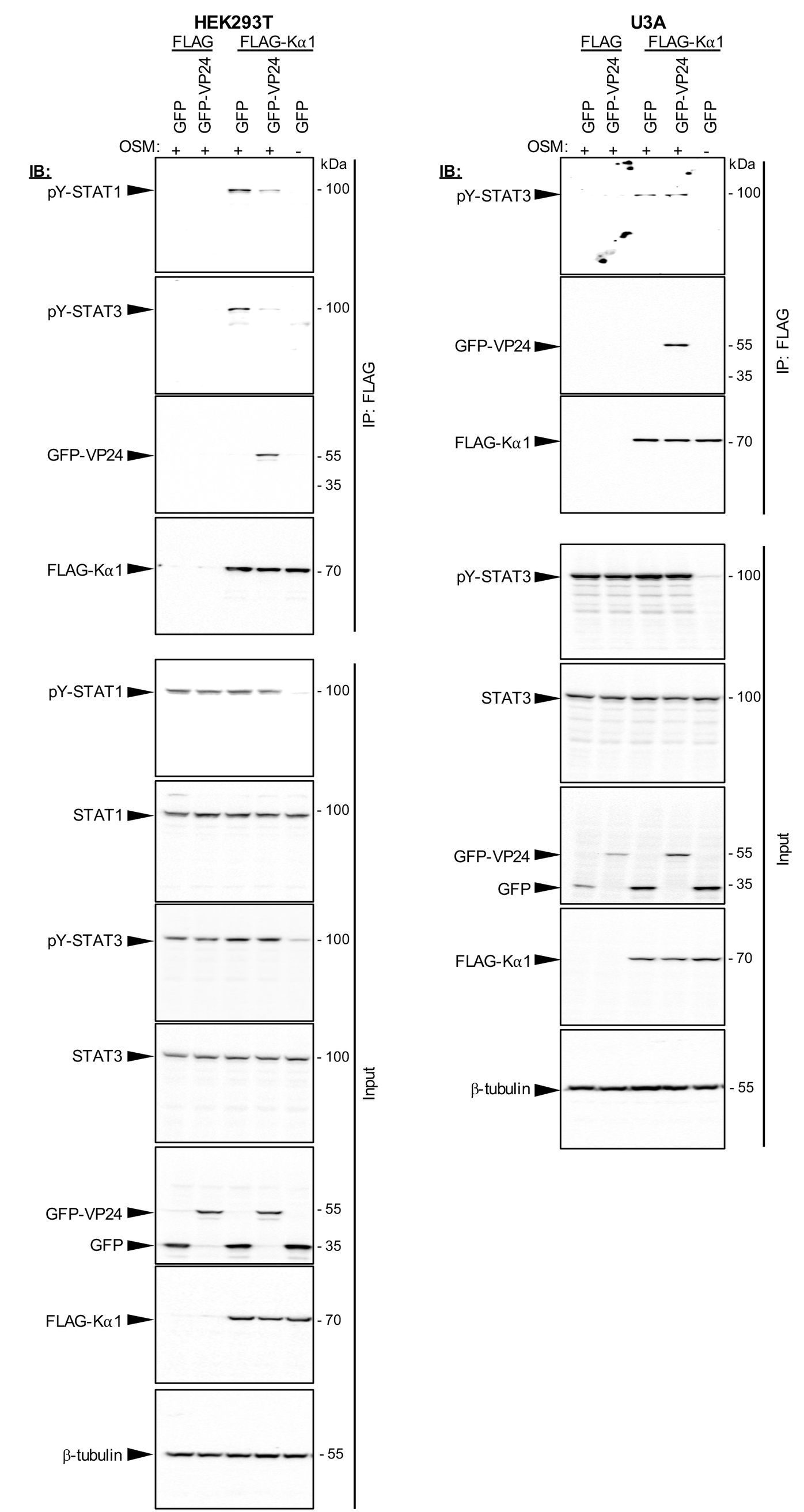

Supplement: S8 Fig — Full images of membranes shown in Fig 5A; arrowheads indicate specific proteins bands. (TIF) [file ppat.1009636.s008.tif]

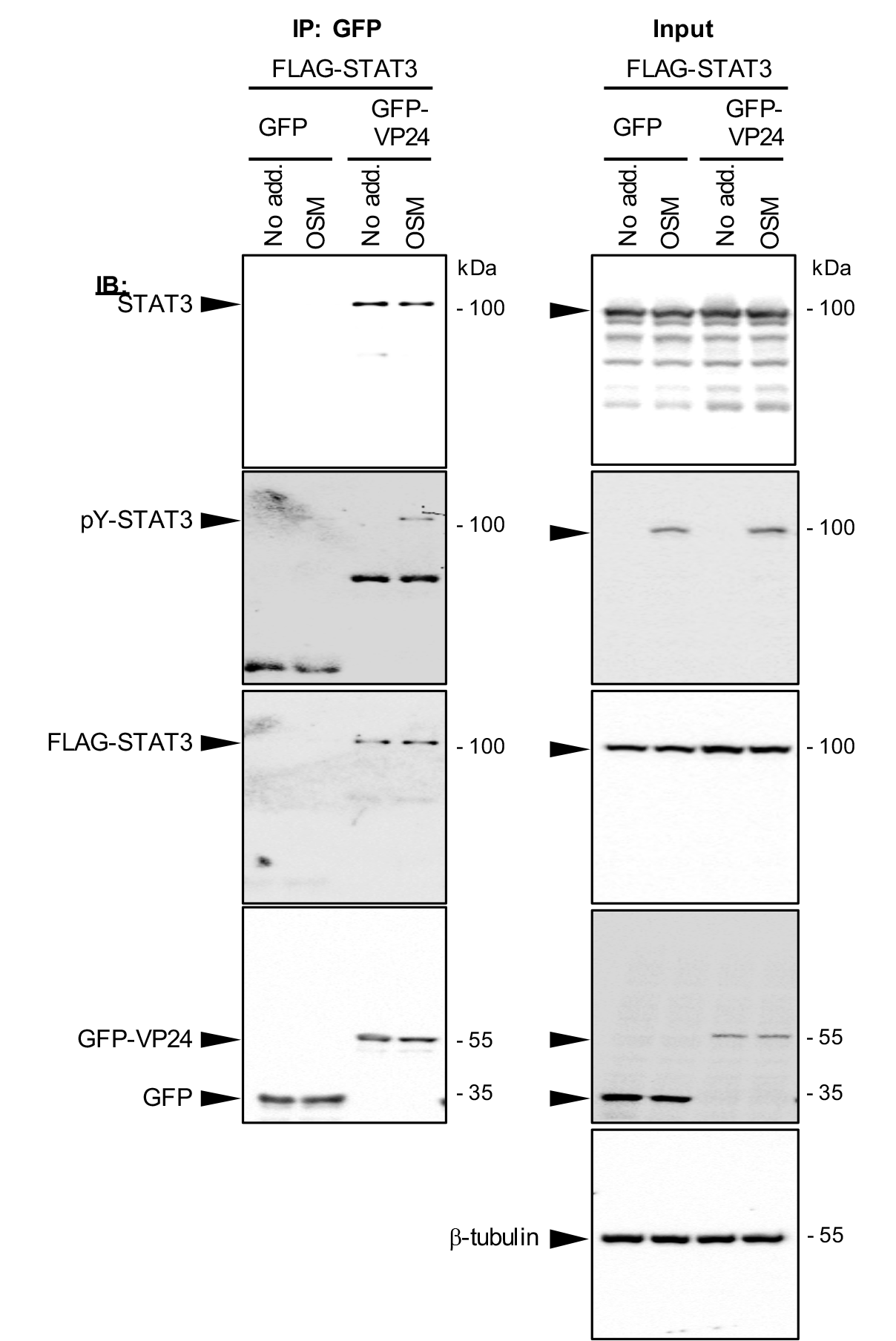

Supplement: S9 Fig — Full images of membranes shown in Fig 7A; arrowheads indicate specific proteins bands. (TIF) [file ppat.1009636.s009.tif]

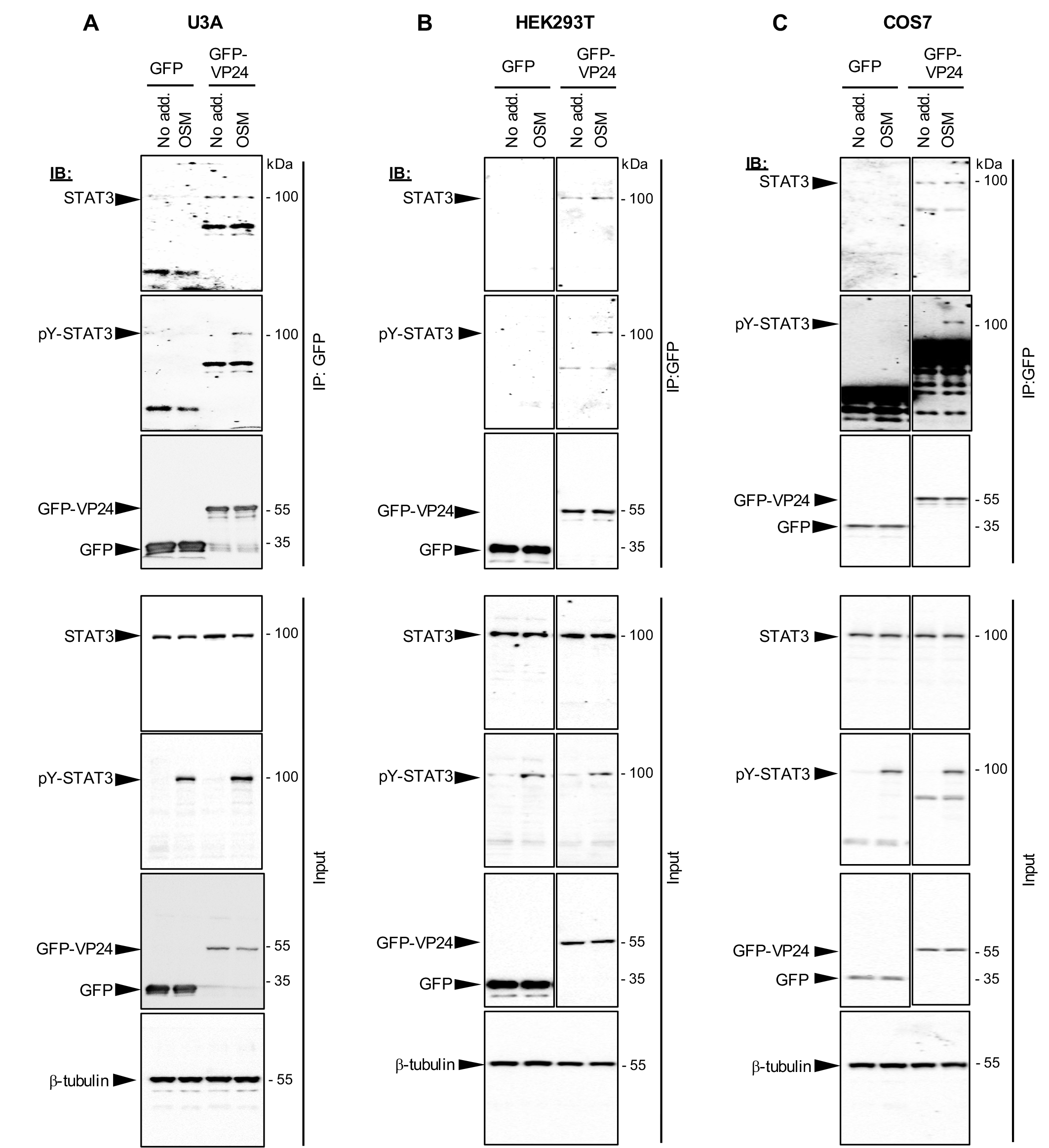

Supplement: S10 Fig — (A, B) Full images of membranes shown in Fig 7B (A) and 7C (B). (C) Results of immunoprecipitation assay using COS7 cells transfected and treated as described for U3A and HEK293T cells in Fig 7B and 7C. Results are representative of 2 independent assays and show data from a single blot with intervening and marker lanes removed. Arrowheads indicate specific proteins bands. (TIF) [file ppat.1009636.s010.tif]

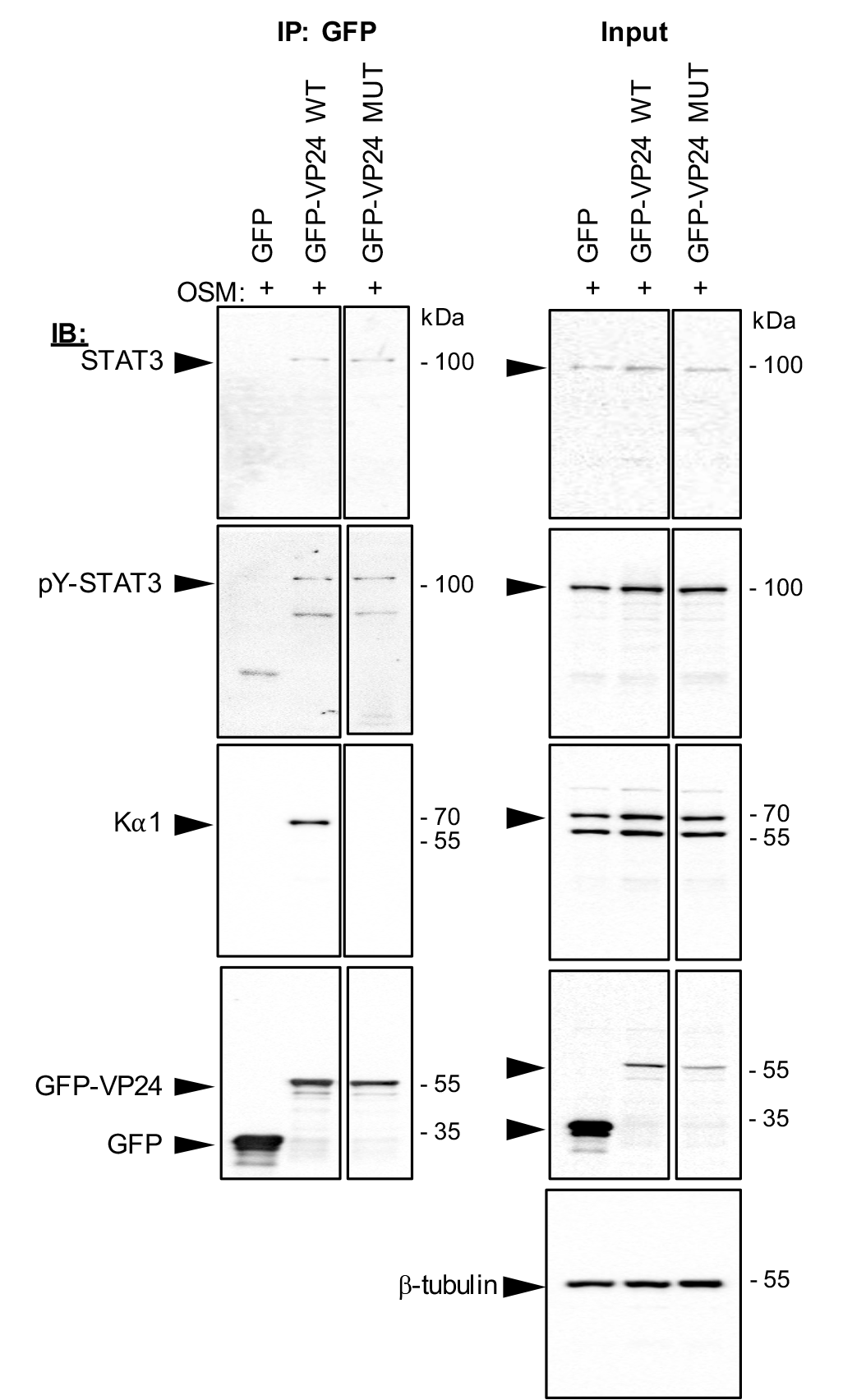

Supplement: S11 Fig — Full images of membranes shown in Fig 8B; arrowheads indicate specific proteins bands. (TIF) [file ppat.1009636.s011.tif]
